# Emerging role of lipid droplets in obscure puffer immune response against *Vibrio harveyi*

**DOI:** 10.1007/s42995-025-00286-w

**Published:** 2025-03-17

**Authors:** Xiaorui Song, Yaxing Yang, Nan Cui, Tianying Lei, Xingkun Jin, Ying Huang, Yan Shi, Zhe Zhao

**Affiliations:** 1 Jiangsu Province Engineering Research Center for Marine Bio-Resources Sustainable Utilization, Hohai University, Nanjing, 210024 China; 2 Department of Marine Biology, College of Oceanography, Hohai University, Nanjing, 210024 China

**Keywords:** Lipid droplets (LDs), *Vibrio harveyi*, *Takifugu obscurus*, Antibacterial activity, Proteomic analyses

## Abstract

As dynamic and functionally active organelles, lipid droplets (LDs) mainly function in lipid anabolism, while recent studies showed that mammalian LDs also actively participated in innate immunity; however, the specific roles and regulation mechanism remain relatively unexplored, and the existing studies were mainly limited to mammals. In the present study, we first found that *Vibrio harveyi*, a serious pathogen in marine environment, could induce LDs accumulation in the liver of obscure puffer *Takifugu obscurus* on the histology, morphology and molecular levels, and the induction mainly conducted by promoting the synthesis of neutral lipids. Moreover, the antibacterial activity of LD proteins was significantly enhanced upon *V. harveyi* stimulation, and showed broad-spectrum characteristic. While the inhibition of LDs formation downregulated the expression of immune-related genes and immune signaling elements, highlighting the potential critical roles of LDs during the bacterial infection. The isolated LDs from obscure puffer liver were examined via proteomic analyses, and the data supported the conservative property of LDs from bacteria to humans, and revealed that numerous innate immune system-related components were enriched on the surface of LDs. These results will deepen the understanding of LDs biology and host immune defense mechanism, shedding light on the new strategies for the development of anti-infective therapies.

**Supplementary Information:**

The online version contains supplementary material available at 10.1007/s42995-025-00286-w.

## Introduction

The lipid droplets (LDs, also known as lipid bodies) are ubiquitous and dynamic organelles found in all types of eukaryotic cells as well as in some prokaryotes (Chen et al. 2020; den Brok et al. 2018; Olzmann and Carvalho 2019). As major lipid storage organelles, LDs are the repository of neutral lipids, and modulate protein segregation, toxic lipids’ elimination as well as cellular signaling (Bosch and Pol 2022; Olzmann and Carvalho 2019; Welte 2015; Zadoorian et al. 2023). In the last two decades, accelerated evidences showed that LDs are a multifunctional organelle and actively participate in various cellular processes, including lipid metabolism, energy homeostasis, membrane trafficking (Welte and Gould 2017), cell signaling (Kagan 2012), as well as inflammation (Pereira-Dutra and Bozza 2021). Especially, recent advancements found that host LDs are active innate immunity players through mediating immunometabolism, immune signaling, and microbial killing (Bosch and Pol 2022; den Brok et al. 2018).

LDs are cellular organelles for lipid metabolism, and predominantly comprise a neutral lipid core (mostly triacylglycerols (TAGs) and cholesteryl esters (CEs)), enveloped by a single layer of phospholipids decorated with a diverse set of proteins (Olzmann and Carvalho 2019; Xu et al. 2018; Zhang and Liu 2019). Distinct from adipocytes, immune cells as well as other non-adipocytes have virtually no or fewer LDs under resting conditions, whereas accumulated LDs were observed when host challenged by different triggers (Bosch et al. 2020; Knight et al. 2018; Monson et al. 2021a, b; Pereira-Dutra et al. 2019; Rabhi et al. 2016). Especially, pathogenic microorganisms’ infection significantly affects LDs formation in the host immune-related cells (Bosch and Pol 2022; den Brok et al. 2018). For example, the number of LDs in dendritic cells (DCs) was significantly increased during infection by *Leishmania amazonensis* or *Nocardia brasiliensis* (Herber et al. 2010). The similar phenomenon was also observed in invertebrate *Aedes aegypti*, and LDs were induced when challenged with *Enterobacter cloacae*, Sindbis, and Dengue viruses (Barletta et al. 2016). In addition, it was reported that pathogen-associated molecular patterns (PAMPs), such as lipopolysaccharides (LPS), mannans, virus mimetics poly I:C, also could induce the accumulation of LDs, as well as the expressions of lipid-related genes in immune and endothelial cells (Bosch et al. 2020, 2021; Czamara et al. 2021; Lei et al. 2019; Monson et al. 2021a, b). Moreover, increased LDs were observed in the infected as well as the adjacent but uninfected cells post killed pathogen stimulation (Chen et al. 2020; Rabhi et al. 2016). While contrary to the traditional views that pathogens could capture and absorb the nutrients and energy from LDs for settlement (Roingeard and Melo 2017), Matthew Knight et al*.* found that *Mycobacterium tuberculosis* can still accumulate lipids in macrophages under the conditions of LDs’ depletion (Knight et al. 2018). Moreover, Monson et al*.* found that after infected with herpes simplex virus 1 and Zika virus, the increased LDs significantly inhibited the virus’s replication ability in mice (Monson et al. 2021a, b), whereas loss of LD mass enhances viral replication (Monson et al. 2018). Thus, host LD accumulation upon infection seems to be a host-driven defense mechanism, which have been coevolved with the host–pathogens interactions.

Currently, LDs’ accumulation was considered as a hallmark of successful immune response, and the immunologic defense mechanism of LDs has attracted increasing attention (Bosch and Pol 2022; Pereira-Dutra and Bozza 2021). For instance, after treated with inhibitors for LDs’ formation, the phagocytic ability, the production of cytokines (such as IFN, IL, etc*.*), as well as antimicrobial peptides of mice macrophages significantly decreased following the decrease of the neutral lipid contents (TAGs and CEs) and the expressions of genes involved in the synthesis of TAGs and CEs (DGAT1, DGAT2, ACSL3, FASN, ACAT, etc*.*) (Castoldi et al. 2020; Knight et al. 2018; Monson et al. 2021a, b). Similarly, various cell types (such as HeLa cells and hepatic cells) with reduced cellular LD contents also showed significantly reduced cytokine production (Monson et al. 2018; Truong et al. 2020), indicating that LDs might play critical role during the immune response against pathogen infection. LD proteins were proved to be vital for LDs organization and function. Numerous LD proteins have been revealed in various cell types and activation conditions by proteomics approaches (Dhiman et al. 2020; Pereira-Dutra and Bozza 2021). An interesting study in invertebrate Drosophila showed that LD proteins extracted from Drosophila embryos could significantly inhibit bacterial growth (Anand et al. 2012). Similarly, LD proteins extracted from LPS stimulated mice liver exhibited extremely strong antibacterial ability (Bosch et al. 2020). Proteomic studies provided broader insights into the functioning of LDs. For example, LD proteome of HeLa CCL2 was found to be modified in the context of *Chlamydia trachomatis* infection (Saka et al. 2015). Comparative proteomics revealed that IFN induced anti-viral proteins viperin, immune-related GTPase (IGTP), and histones are specifically enriched on the surface of LDs, and intrinsic immune signaling pathway elements, such as STING, TBK1, IRAK1, and TRAF6, are all co-localized on the LDs’ surface (Bosch et al. 2020; Crosse et al. 2021; Hinson and Cresswell 2009; Saitoh et al. 2011). These studies indicate that LDs functions directly and actively during the innate immune response. However, the specific roles and regulation mechanism of LDs in innate immunity remain relatively unexplored, and the existing studies were mainly limited to mammals. Further exploration is urgently needed to investigate this antibacterial effect in different pathogen infection models.

Fish has long been one of the research models due to the high diversity of species and the unique evolutionary status. *Vibrio harveyi* is a widely regarded pathogenic bacteria in marine aquaculture environment, even posing a great threat to human health (Mohi et al. 2010; Zhang et al. 2020). Here, we employed the important aquaculture fish, *Takifugu obscurus*, which has relatively small and compact genomes compared to other vertebrates (Kang et al. 2020), with the aim of unraveling the functional and regulatory mechanism of LDs on anti-Vibrio immunity.

## Materials and methods

### Fish and microbes

Obscure puffer *T. obscurus* was obtained from an aquatic farm (Nantong, China), and maintained in re-circulating water (200 L, salinity: 4) at 20–25 °C. The average weight of the fish is ~ 50 g, and daily baiting amount is 3–10% of fish’s body weight. The day before the experiments, fish were fasted overnight (~ 16 h).

The bacterial strains used in this study *Vibrio harveyi* (E385) and *Staphylococcus aureus* (CMCC(B) 26003) were obtained from the Institute of Microbiology, Hohai University, and cultured using Luria Bertani medium with salinity at 20 at 30 °C and 37 °C, respectively. After centrifugal separation, the precipitates were washed and diluted in sterile physiological saline (0.75% NaCl) to 1.0 × 10^6^ colony-forming units (CFU)/mL. The killed bacteria were obtained via heated at 100 °C for 5 min.

### Immune stimulation and samples’ collection

Three independent experiments were conducted (named 1, 2, and 3 thereafter) for the immune stimulation, and 108 fish were employed in total. For the experiment 1, 27 fish were randomly divided into three groups. Each fish in first and second group was intra-peritoneally (i.p.) injected with 100 μL of live and heat-killed *V. harveyi*, respectively, and the third group was i.p. injected with an equal amount of sterile physiological saline (0.75% NaCl). The liver and head kidney samples were collected at 24 h as well as 48 h, respectively.

For the experiment 2, 36 fish were randomly divided into four groups. Each fish in the first group was challenged by i.p. injection with 100 μL of live *V. harveyi* (OD600 = 0.3). Similarly, each fish in the second and third group was i.p. injected with 100 μL of *V. harveyi* OD600 = 0.6 and 1.0, respectively. While the fish in the control group was injected with an equal volume of sterile physiological saline. The liver and head kidney samples were collected at 24 h.

Thirty-six fish were employed for the experiment 3. After sampled at 0 h, other 27 fish were received an i.p. injection with 100 μL of *V. harveyi* (OD600 = 0.6) and live tissue was subsequently sampled at 24 h, 3 d, and 7 d post-stimulation.

Three parallel samples were pooled as one biological replicate, and there were three biological replicates for each time point in all the experiments.

### Purification of lipid droplet proteins

Lipid droplets were isolated as previous described with minor modification (Bosch et al. 2020; Ding et al. 2013). Briefly, after washed with sterile physiological saline, the liver was homogenized (the ratio of wet weight (g): homogenization buffer (mL) = 1: 3). After centrifuged at 500*g* at 4 °C for 10 min, the post-nuclei supernatant of liver homogenates (~ 2.5 mL) was collected. The mixture made by mixing the collected supernatant with the same amount of sucrose solution (2.5 mol/L, Sangon Biotech, China) was layered at the bottom of a sucrose step gradient of 25%, 15%, 10%, and 5% (W/V), with an additional equal amount top layer of homogenization buffer, and centrifuged at 12,000 *g* for 1 h at 4 °C (Beckman, Germany). After centrifugation, four fractions from the top of each gradient were sampled for western blot analysis. According to LD’s biological property (low density), the top layer of the gradient (5%) was recovered and concentrated at 16,000 *g* for 10 min at 4 °C. After adding four volumes of pre-cooled acetone, the samples were maintained at − 20 °C for 48 h, and re-centrifuged at 12,000 *g* for 10 min at 4 °C, the pellets were washed with pre-cold acetone, vacuum freeze-dried to powder, and re-dissolved in Tris-HCl (10 mmol/L, pH 7.5).

### Silver staining analysis

For the silver staining, LD proteins extracted from the liver of obscure puffer were first separated via SDS-PAGE. The gel was fixed, and incubated in a sensitizer solution at room temperature for 30 min. After extensively washed with ddH_2_O, the gel was incubated with silver staining solution and developing solution (Beyotime, China), successively. Finally, the reaction was stopped when the bands appeared.

### Western blot

The liver tissues were homogenized by adding 500 μL of RIPA buffer (Beyotime, China). After incubation on ice for 30 min, the homogenization buffer was centrifugated, and the supernatant was collected. After separated by SDS-PAGE, the proteins were transferred onto the PVDF membranes, and then incubated with primary antibodies (rabbit polyclonal anti-PLIN2 (1:1000; 381796, Zenbio, China), rabbit polyclonal anti-PLIN3 (1:800; 123356, Zenbio, China), GAPDH (1:2000, 380626, Zenbio, China)), and secondary antibodies (Goat Anti-Rabbit IgG H and L (1:5000, 511203, Zenbio, China)), successively. After three times extensive washing, the signals were detected by dipping in the ECL buffer (Vazyme, China) in the dark for about 5 min, and visualized using Image Quant LAS4000 (Protein Simple, FluroChem E, USA).

### Quantitative proteomics analysis

To explore the dynamic changes of liver LDs post *V. harveyi* stimulation, LD proteins extracted from control group and *V. harveyi* stimulated group were digested with trypsin and subjected to the proteomic analysis using the 4D-label-free-based quantitative method, which was carried out by Jingjie PTM Biolabs Inc. (Hangzhou, China). Briefly, short peptides were obtained by purified LDs proteins digestion, separated, and analyzed. The resulting DIA data were searched against Takifugu_rubripes_31033_PR_20230529.fasta (51,079 entries). Excision on N-term Met and carbamidomethyl on Cys were specified as fixed modification. False discovery rate (FDR) was adjusted to < 1%. After repeatability evaluation, differential proteins were identified, annotated, and analyzed according to previous reports (Ding et al. 2023).

### Transmission electron microscopy (TEM)

The liver of the *T. obscurus* were surgically isolated, scissored excised, and washed with filtrated saline (0.85% NaCl). After chopped to 1 cm^3^ species, samples were fixed for 24 h in 2.5% glutaraldehyde (Sangon Biotech, China), sectioned using an ultramicrotome (Leica, Germany), and secondary fixed with osmium tetraoxide. Finally, imaging was performed on a Hitachi 7700 (Tokyo, Japan) at 80 kV.

### Antibacterial assays

Colony forming assays and bacterial inhibition assays were conducted to examine the antibacterial capability of LD proteins. Bacterial culture (100 μL, 1.0 × 10^6^ CFU/mL) was mixed with 75 μg or 175 μg of LD proteins (isolated from control and Vh groups, respectively, and dissolved in Tris-HCL) and incubated at room temperature for 2 h. Equivalent Tris-HCL was used as control. Mixture (200 μL in total) diluted with LB medium by tenfold were distributed into each well, and incubated at 37 °C (Bioscreen, Finland) to assay bacterial growth curve meter. The growth of *V. harveyi* (E385) and *S. aureus* (CMCC (B) 26003) at OD 600 nm was tested every 1 h from 13 to 23 h. Each group was repeated for three times.

The antibacterial activity of LD proteins was also measured using a CFU assay as previous reports (Anand et al. 2012; Bosch et al. 2020). Briefly, dilutions (1:3000) were spread in triplicate on LB-agar plates and bacteria colonies were counted as CFU/mL on plates after culturing at 37 °C overnight.

### Quantitative real-time PCR (qRT-PCR)

For determine the expressions of target genes, total mRNA was extracted from tissues using the Trizol reagent (Invitrogen, USA), and cDNA templates were reversely transcribed using about 1 μg of mRNA. qRT-PCR was conducted using the TB Green® Premix Ex Taq™ II FAST qPCR (Takara, China) and detected by the LightCycler® 96 qRT-PCR System (Roche, Switzerland). Relative expressions of each gene were normalized to the internal reference RS18, and exhibited as fold-change compared to control group. All the primers are provided in Supplementary Table S1, and all the data were calculated by the 2 ^−ΔΔCT^ method.

### Determination of triglycerides (TAGs) and cholesterol esters (CEs) contents

Triglycerides (TAGs) and cholesterol esters (CEs) contents were determined by the GPO-PAP method test kit (Elabscience, China). About one gram of fresh liver and kidney tissue was collected, rinsed with 1 × PBS at 4 °C, and, homogenized [the ratio of wet weight (g): pre-cooled isopropanol (mL) = 1: 9] on ice for 10 min. After centrifuged at 10,000 *g* for 10 min under 4 °C, the supernatants were measured at 510 nm to determine the contents of TAGs and CEs as kit’s instruction, and the experiments were performed in triplicates. Both contents were represented as μmol of per gram wet weight of tissues.

### Treatment of fish with lipid droplet formation inhibitor T863

DGAT1 inhibitor T863 was used to inhibit the lipid droplet formation (Knight et al. 2018). For the experiment, 30 fish were randomly divided into three groups. Each fish in the first group received an i.p. injection with 100 μL of *V. harveyi*. For the second group, each fish was pretreated with 100 μL of *V. harveyi* and T863 (2.5 μmol/L, MCE, USA) was added 1 h later. Fish injected with 100 μL sterile physiological saline (0.75% NaCl) were considered as control group. The liver and head kidney samples were collected at 24 h.

### Statistics

All the above experiments were conducted in triplicate. Statistical analyses were carried out by Student’s t test. *P value < 0.05 was considered as significance.

## Results

### *V. harveyi* stimulation significantly induced the LDs’ accumulation in the liver of obscure puffer

**Fig. 1:**
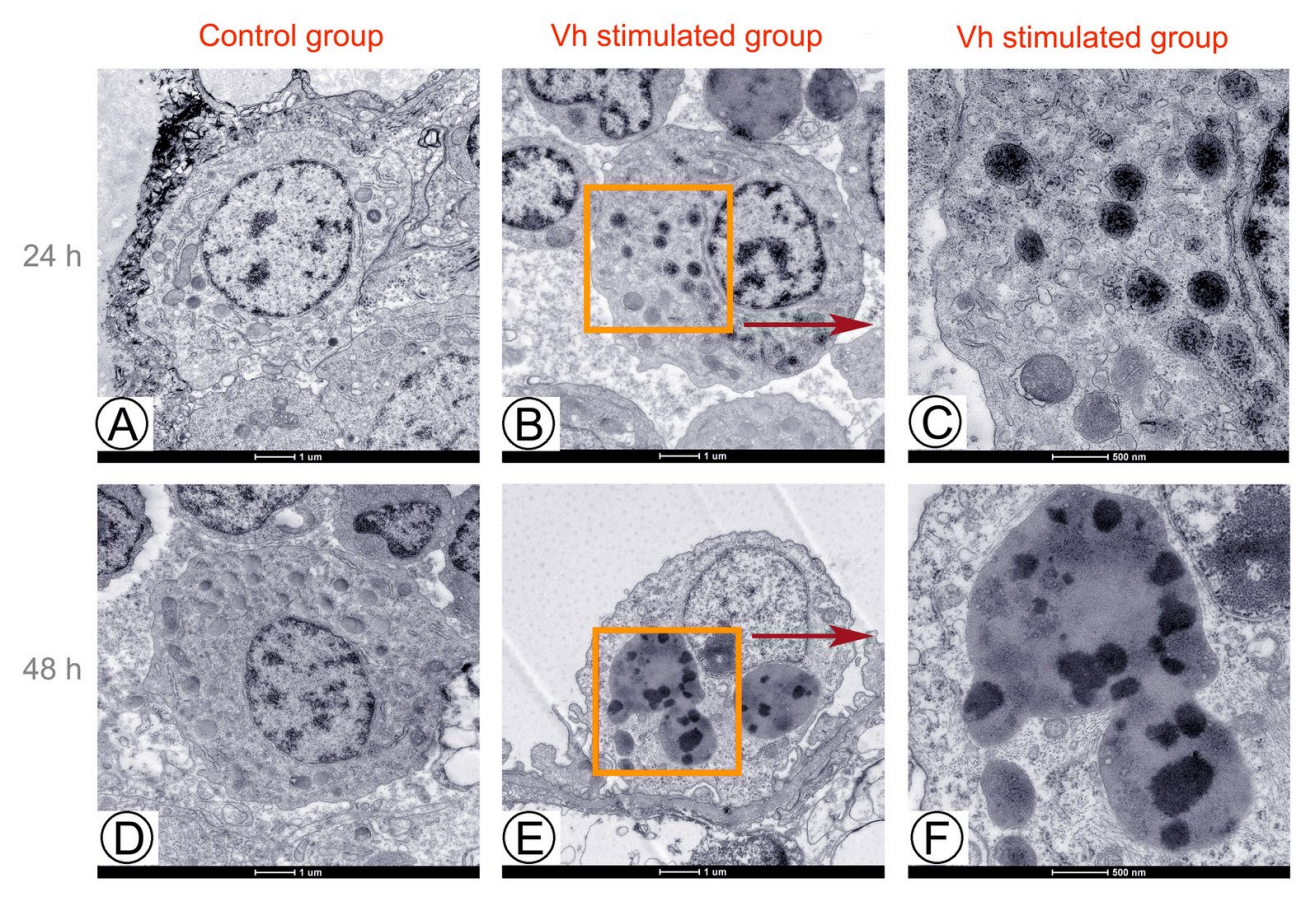
Increasing cellular LD numbers in TEM images of *Vibrio harveyi* stimulated liver. **A, D** TEM images revealed fewer LDs in the liver from control group at 24 h (**A**) and 48 h (**D**), respectively; **B, C** TEM images revealed increased cellular LD numbers in the liver at 24 h post *V. harveyi* stimulation (**B**), and the boxed area was enlarged in the (**C**). **D, E** TEM images revealed increased cellular LD numbers and size in the liver at 48 h post *V. harveyi* stimulation (**D**), and the boxed area was enlarged in the (**E**). Scale bar = 1 μm (**A, B, D, E**); Scale bar = 500 nm (**C, F**)

To investigate whether bacteria stimulation could induce LDs accumulation in fish, obscure puffer was stimulated with live *V. harveyi* by i.p. injection. We selected liver as target tissue which modulates the systemic immune response. Perilipins are kind of typically LDs’ surface proteins and generally used to measure the LDs’ formation (Sun et al. 2023; Sztalryd and Brasaemle 2017; Zhang and Liu 2019). In the experiment 1, compared with the control group, PLIN2 expression was both significantly induced by live *V. harveyi* stimulation at 24 and 48 h (Supplementary Fig. S1A). To further confirm the induction of *V. harveyi* on the LDs’ accumulation, heat-killed *V. harveyi* was also employed, and the expression patterns of PLIN2 exhibited the similar enhancement post-stimulation (Supplementary Fig. S1B), indicating that *V. harveyi* stimulation could induce LDs accumulation in obscure puffer, which should be a host-driven mechanism. Next, the effects of increasing bacterial concentrations on LDs’ accumulation were assayed, and the expression levels of PLIN2 showed significant increasement along with the bacteria concentration increase, and exhibited the highest expressions at the highest bacterial concentration of OD = 1 (Supplementary Fig. S1C), indicating that LD accumulation induced by *V. harveyi* infection is in dose-dependent manner. In the time-course analysis, compared to the control group, PLIN2 showed the highest levels at 24 h, and also higher at three days post-stimulation, while returned to the normal level at seven days post-stimulation (Supplementary Fig. S1D). The liver sections from obscure puffer were examined via transmission electron microscopy (TEM). A clear increase in the presence of large LDs as well as an increase in small LDs were observed in the macrophage of *V. harveyi* infected liver both at 24 (Fig. 1B, C) and 48 h (Fig. 1E, F) post-stimulation, which was absent in the liver sections from control group (Fig. 1A, D). Specially, LDs aggregation seems to be stimulated by *V. harveyi* at 48 h post-stimulation (Fig. 1E, F). We also examined liver sections taken from killed *V. harveyi* infection. Similarly, increased LDs’ numbers and size were visualized in the macrophage both at the 24 and 48 h, respectively (Supplementary Fig. S2). Above results suggest that *V. harveyi* stimulation could induce the newly formation and expansion of the original LDs, and this phenomenon should result from host-driven mechanism.

### *V. harveyi* stimulation induced lipid droplets’ formation by promoting the synthesis of neutral lipids

**Fig. 2:**
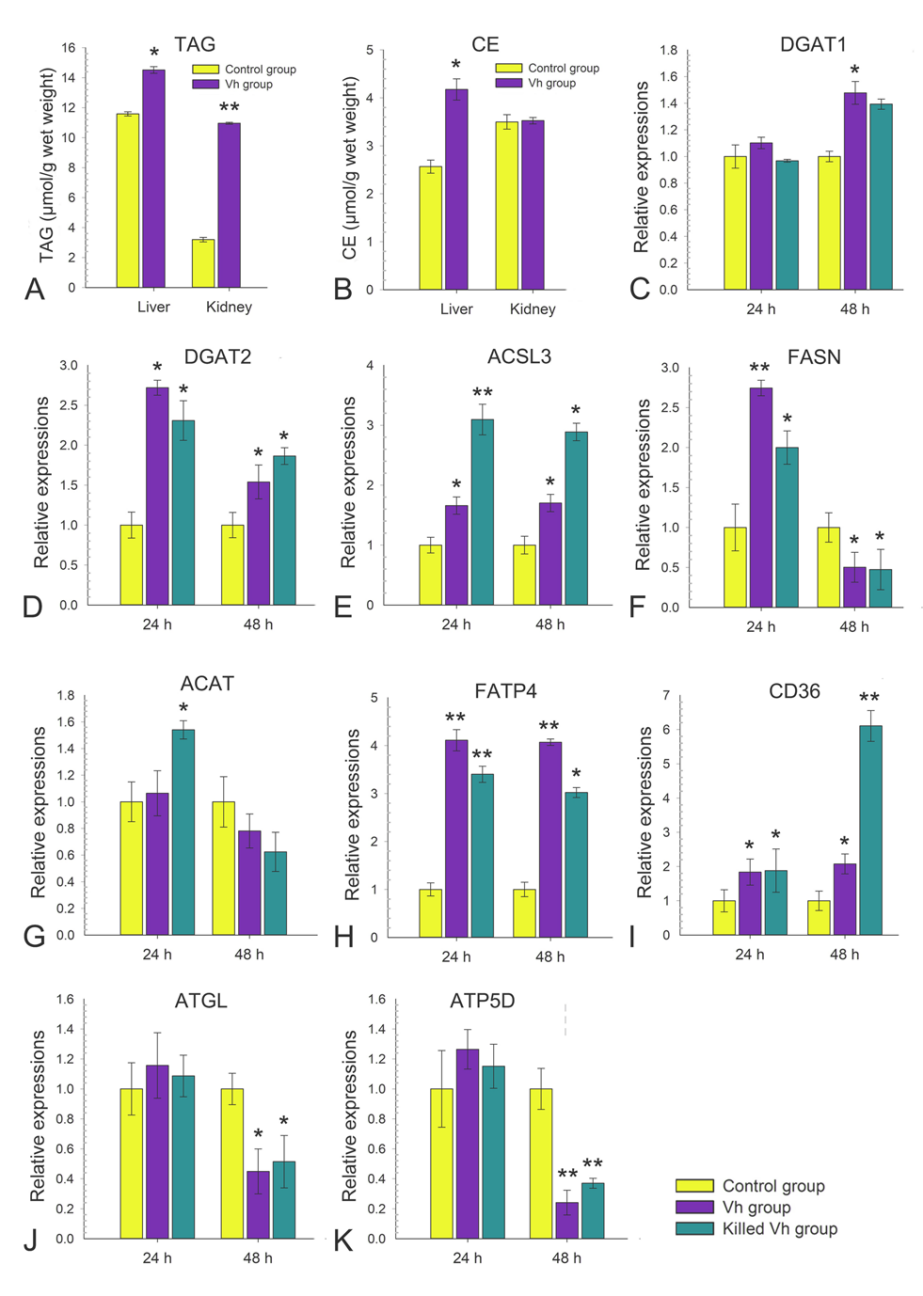
*Vibrio harveyi* stimulation induced lipid droplets’ formation by promoting the synthesis of neutral lipids. **A** The contents of triglycerides (TAGs) in liver and kidneys at 24 h post *V. harveyi* stimulation. **B** The contents of cholesterol esters (CEs) in liver and kidneys at 24 h post *V. harveyi* stimulation. **C–K** qRT-PCR analysis of expression of genes involved the synthesis of neutral lipids (DGAT1, DGAT2, ACSL3, FASN, ACAT, FATP4, CD36, ATGL, and ATP5D) in control, *V. harveyi* and killed *V. harveyi* stimulated groups. Data are mean ± SD, n = 3 biological replicates. Two-tailed unpaired t tests. **P < 0.001; *P < 0. 05

TAGs and CEs are the core neutral lipids of LDs, and thus, their biosynthesis is associated with LDs’ accumulation. Hence, the contents of TAGs and CEs in liver and head kidney post *V. harveyi* stimulation were assayed. Upon *V. harveyi* stimulation, TAGs exhibited a sharp increase reaching to 10.96% of wet weight in head kidney at 24 h, while only 3.19% in control group (*P* < 0.05, Fig. 2A). TAGs also showed a considerable increase in liver, from 11.59% in control group to 14.51% in Vh group at 24 h (*P* < 0.05, Fig. 2A). Likewise, CEs’ contents also increased from 2.57% in control group to 4.18% in Vh group at 24 h (*P* < 0.05), while no significant differences were detected between control and Vh group in head kidney (Fig. 2B).

Next, we examined the regulation of TAGs and CEs metabolism during the stimulation. Diacylglycerol O-acyltransferases (DGAT, including DGAT1 and DGAT2) are critical enzymes involving de novo TAG synthesis. Compared with the control group, live *V. harveyi* stimulation increased DGAT1 mRNA levels by approximately 1.5-fold post-stimulation at 24 h, while there was no significant difference between killed *V. harveyi* stimulation and control group (Fig. 2C). For the DGAT2, the mRNA levels were both significantly upregulated by approximately 2–threefold and 1.5–twofold post-stimulation at 24 and 48 h either with live or killed *V. harveyi*, respectively (*P* < 0.05, Fig. 2D). Fatty Acid Synthase (FASN) and acyl-CoA synthetase long-chain family member 3 (ACSL3) which involves the de novo synthesis of glycerolipids through the Kennedy pathway. The mRNA levels of ACSL3 at 24 and 48 h were both significantly upregulated by 3.1- and 2.89-fold with killed *V. harveyi* (*P* < 0.05, Fig. 2E), while mRNA transcripts of FASN only increased by 2.7- and twofold at 24 h both in the live and killed *V. harveyi* stimulation group, respectively (*P* < 0.05, Fig. 2F). Acyl coenzyme A-cholesterol acyltransferase (ACAT) is a kind of Acyl-CoA participating in cholesterol esterification. The mRNA transcripts of ACAT only increased at 24 h by 1.54-fold in the killed *V. harveyi* stimulation group (*P* < 0.05, Fig. 2G). Fatty acid transport proteins (FATPs) and fatty acid translocase (CD36) participate in the fatty acid uptake, and thus contribute to LD accumulation. The mRNA levels of FATP4 and CD36 both significantly upregulated post-stimulation at 24 and 48 h (*P* < 0.05, Fig. 2H, I). Adipose triglyceride lipase (ATGL) is critical enzyme for neutral lipid degradation, and ATP5D is a subunit of adenosine 5’-triphosphate (ATP) synthase, mediating the interaction of LDs and mitochondria. Both live and killed *V. harveyi* stimulation significantly downregulated the mRNA transcripts of ATGL and ATP5D at 48 h, whereas *V. harveyi* stimulation did not alter their expressions at 24 h (Fig. 2J, K). These results correlated well with the accumulation of LDs post *V. harveyi* stimulation, and suggesting that *V. harveyi* stimulation induced lipid droplets’ formation mainly by promoting the synthesis of TAGs and CEs.

### *V. harveyi* stimulated LDs displayed enhanced antibacterial activity

To further decipher the potential functions of LDs, LD proteins were extracted from the isolated LDs of obscure puffer liver according to the previous studies (Ding et al. 2013; Sun et al. 2023). Liver LDs were isolated by differential centrifugation with sucrose density gradient (Supplementary Fig. S3A), and the LD proteins were extracted via acetone precipitation method. Western blot analysis revealed that strong bands of the PLIN2 and PLIN3 were observed in the fraction that located on the top layer of the gradient (5%), but almost no detectable in other fractions except for weak band of PLIN3 in 15% and 25% gradients (Supplementary Fig. S3B). Three independent LD proteins extracted from control (C1-C3, control LDs) and *V. harveyi* stimulated groups (E1-E3, Vh-LDs) were quantified, and the results revealed that the protein patterns of LD proteins were quite clear and distributed normally (Supplementary Fig. S3C), which was basic consistent to the previous reports (Bosch et al. 2020; Sun et al. 2023). The concentrations of LD proteins were also determined and the results showed that it was suitable and adequate for functional and proteomic analysis (Supplementary Fig. S3D).

**Fig. 3:**
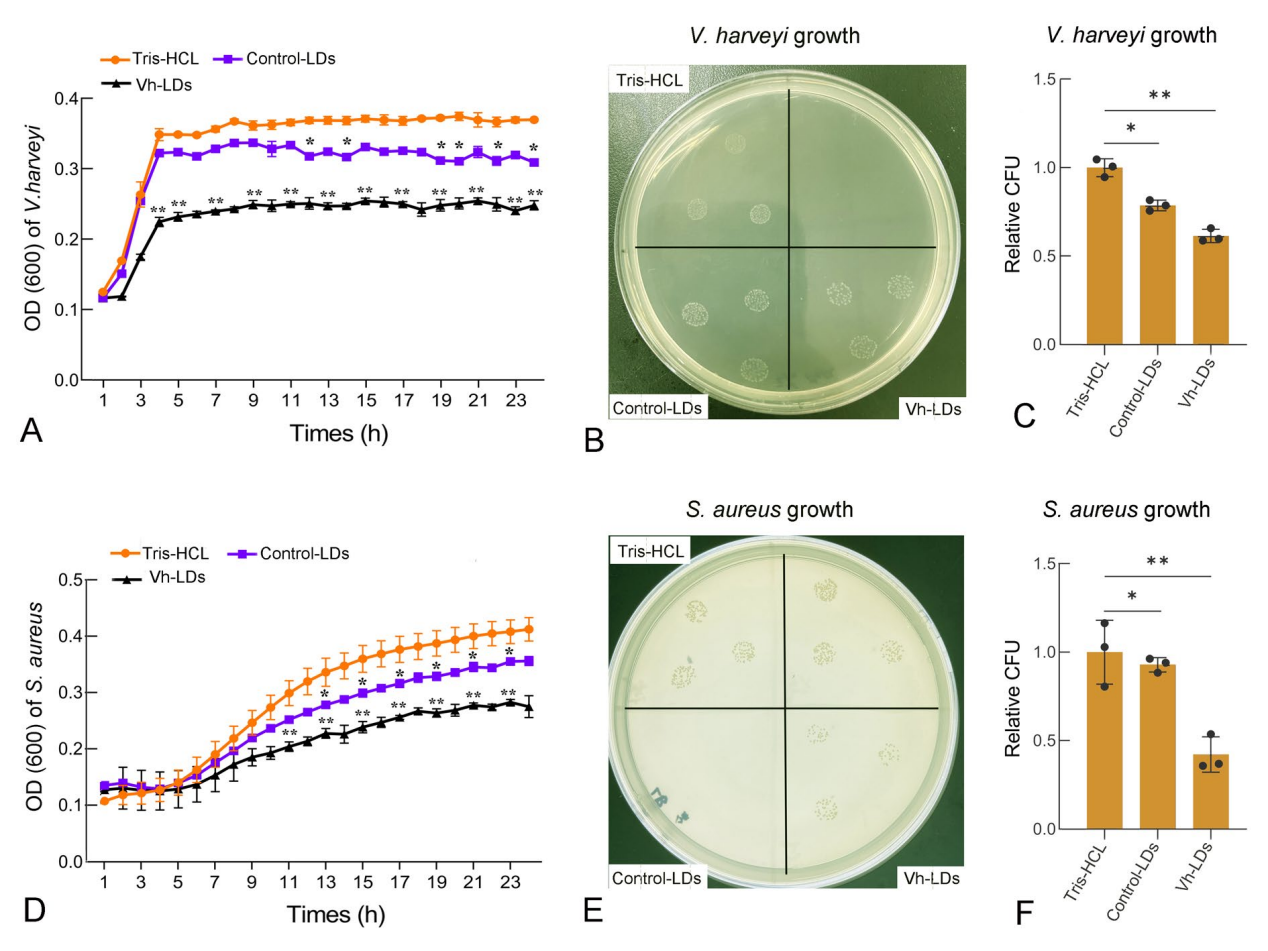
The antibacterial capacity assay of LD proteins. **A** *Vibrio harveyi* were cultured for indicated times in either growth media with Tris-HCL (red) or in media containing 175 μg of proteins from control LDs (blue), or *V. harveyi* stimulated LDs (black). At indicated times, the OD600 of *V. harveyi* growth was measured and quantified relative to the control group. **B** *Staphylococcus aureus* were cultured for indicated times in either growth media with Tris-HCL (red) or in media containing 175 μg of proteins from control LDs (blue), or *V. harveyi* stimulated LDs (black). At indicated times, the OD600 of *S. aureus* growth was measured and quantified relative to the control group. **C, D** After 2 h, serial dilutions of the culture were plated in soft agar, and the number of growing colonies (colony-forming units, CFU) was counted and referred to the control condition (representative of three fish per condition). Data are mean ± SD, *n* = 3 biological replicates. Two-tailed unpaired *t* tests. **P < 0.001; *P < 0. 05

To test antibacterial activity of LD proteins, the bacteria growth curve assay was performed. The results showed that control LDs showed slightly inhibitory effects on the grow of Gram-negative *V. harveyi* and Gram-positive *S. aureus* from 12 to 23 h compared with Tris–HCL group (*P* < 0.05, Fig. 3A, D), while Vh-LDs clearly restrained the growth of *V. harveyi* from 5 to 23 h compared with the Tris-HCL incubation group (*P* < 0.01, Fig. 3A). Similarly, the OD 600 value of *S. aureus* in Vh-LDs treatment group was significantly lower than that in Tris-HCL group at 11–23 h post-treatment (*P* < 0.01, Fig. 3D).

In the traditional plate assay, the average CFU number for Gram-negative *V. harveyi* in Vh-LDs group was 61, which was significantly reduced compared with the control LDs (79) and Tris-HCL (100) group (*P* < 0.05, Fig. 3B, C). For the Gram-positive bacteria *S. aureus*, the average number of CFU in Vh-LDs group was 37, which was significantly reduced compared with the CFU in control (83) and Tris-HCL (89) incubation groups (*P* < 0.05, Fig. 3E, F). These results collectively indicated that LD proteins isolated form obscure puffer liver possess antibacterial activity, which could be enhanced by *V. harveyi* stimulation. In addition, antibacterial activity of Vh-LDs exhibited broad-spectrum both against Gram-negative and Gram-positive bacteria.

### Proteomic analyses of isolated LDs from obscure puffer liver

**Fig. 4:**
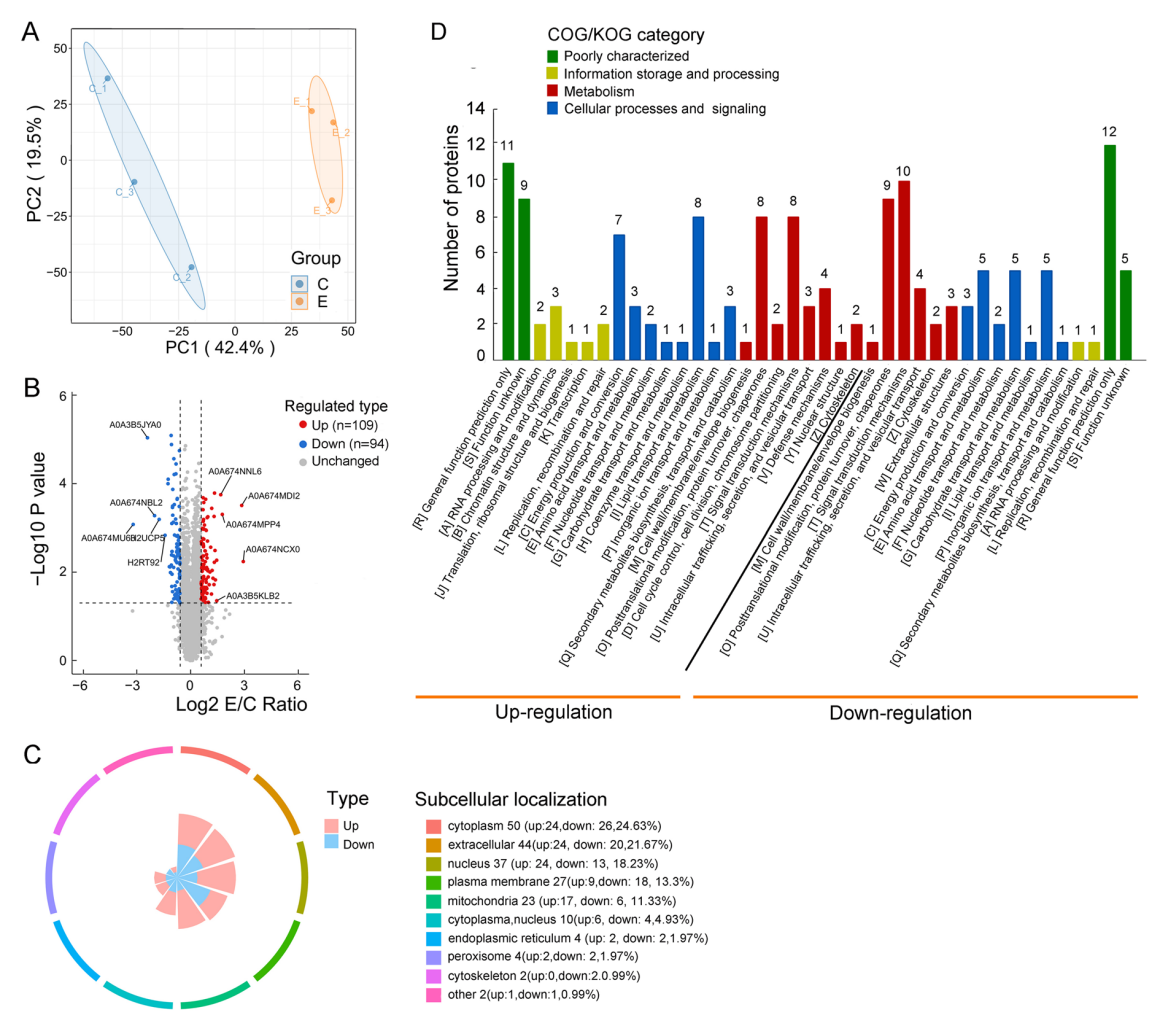
Quantitative proteomics revealed dynamic changes of LD proteins post *Vibrio harveyi* stimulation. **A** The principal component analysis (PCA) at the proteomics level for a total of six liver LD samples of the control and Vh groups. **B** Differentially expressed proteins were displayed by volcano plots. The vertical lines correspond to Log_10_ *P* value, and the horizontal line represents the Log_2_ E/C Ratio. Upregulation of protein expression in Vh group is shown in red, while downregulation is shown in blue. Unchanged proteins were indicated as gray. **C** Subcellular localization analysis of differentially expressed proteins. The pink indicated the upregulated proteins, while the blue indicated the downregulated proteins. **D** KOG classifications of differentially expressed proteins. The ordinate denotes the number of proteins in each KOG category

**Fig. 5:**
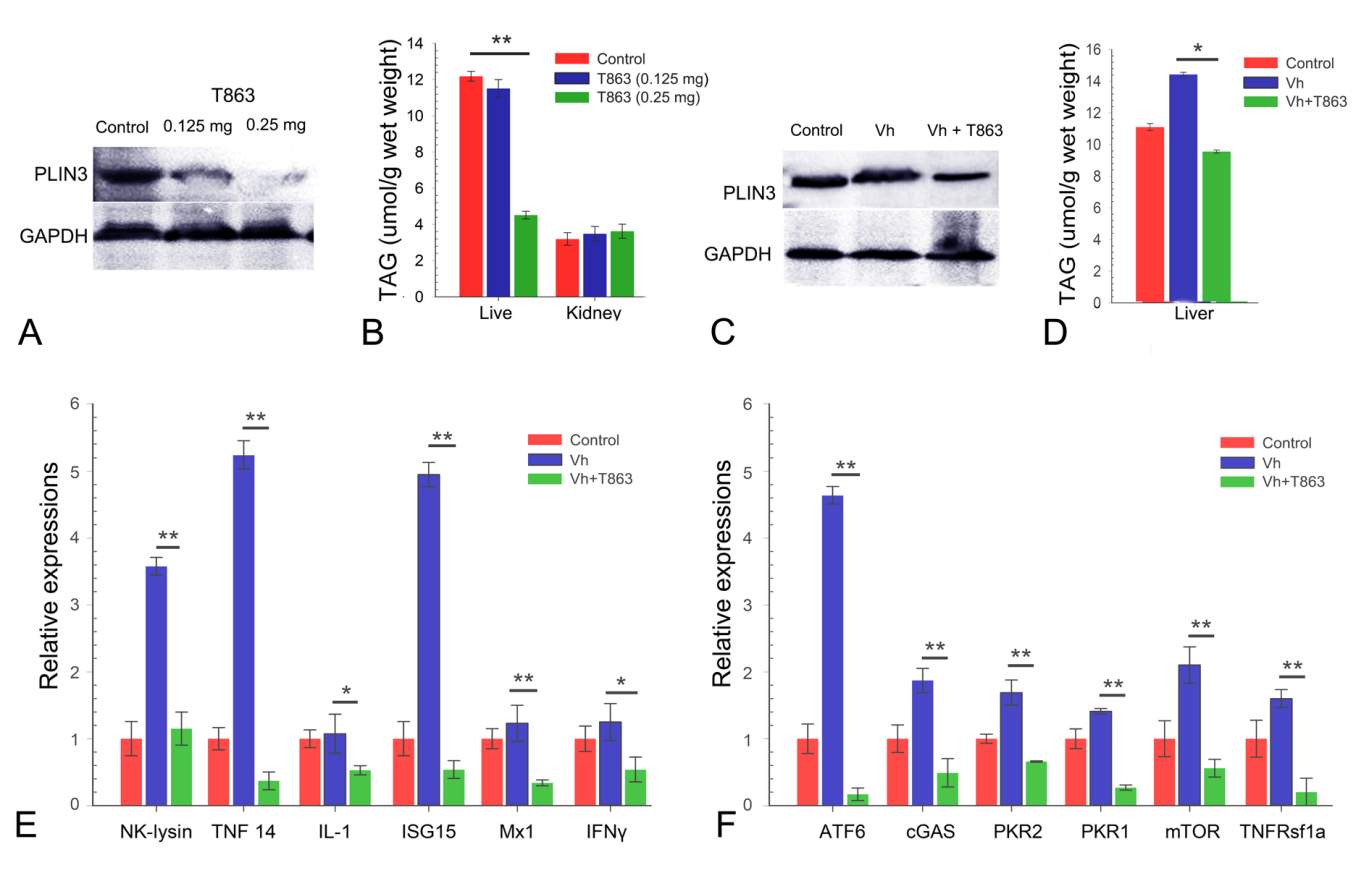
The inhibition of LDs formation decreases PLIN3 expressions, TAG contents, and mRNA levels of immune-related genes. **A** Treatment with DGAT1 inhibitor T863 significantly decreased triglycerides (TAGs) contents in liver under the higher dose (0. 25 mg). **B** Treatment with DGAT1 inhibitor T863 decreased LD-resident protein PLIN3 expression in liver both under the lower dose (0.125 mg) and higher dose (0. 25 mg). **C** Pre-treatment with DGAT1 inhibitor T863 reduced the contents of liver triglycerides (TAGs) at 24 h in liver post *Vibrio harveyi* stimulation. **D** Pre-treatment with DGAT1 inhibitor T863 attenuated the expressions of LD-resident protein PLIN3 at 24 h in liver post *V. harveyi* stimulation. **E, F** Pre-treatment with DGAT1 inhibitor T863 significantly downregulated the mRNA levels of immune-related genes in liver. Scale bar = 1 μm (**A, B, D, E**); Scale bar = 500 nm (**C, F**)

The entire raw data for six proteomes from control and *V. harveyi* stimulated group showed a total of 30, 014 peptides, 5266 identified proteins, and 5217 comparable proteins were quantified and annotated in obscure puffer liver LDs (Supplementary Figs. S4, S5A, B), of which 203 were differentially expressed between the control and Vh groups, including 109 upregulated and 94 downregulated (fold-change > 1.5 and *P* < 0.05, Supplementary Fig. S5C, Fig. 4A, B). The PCA analysis showed that the two groups of control (C1, C2 and C3) and *V. harveyi* stimulated (E1, E2, and E3) were relatively separated from each other (Fig. 5A). It was revealed that the significantly expressed proteins mostly distributed on cytoplasm proteins (50, 24.63%), extracellular proteins (44, 21.67%), and nucleus proteins (37, 18.23%) (Fig. 4C). Next, the COG/KOG results showed that *V. harveyi* markedly upregulated proteins, excluding the poorly characterized group (20 upregulated and 17 downregulated proteins), were mainly involved in lipid transport and metabolism, posttranslational modification, protein turnover, chaperones, and signal transduction mechanisms (Fig. 4D, Supplementary Table S2). For further analysis, these differential proteins were divided into four clusters based on the changing tendencies (Q1: 24; Q2: 70; Q3: 90; Q4: 19), and differentially expressed proteins in each cluster were then hierarchically clustered (Supplementary Fig. S6). It is worth noting that the proteins with the most significant difference in Q4 cluster were involved in icosanoid metabolic process, which mainly contribute the production of essential fatty acid as well as inflammatory mediator eicosanoid (Pereira-Dutra and Bozza 2021). Many functional enzymes, including ATP-citrate synthase (ACLY), Enoyl-CoA Delta Isomerase 1 (ECI1), Diacylglycerol Acyltransferase, Trimethyllysine dioxygenase, 15-hydroxyprostaglandin dehydrogenase (HGPD), 17 β-hydroxysteroid dehydrogenase (17β-HSDs), and ceramide transfer protein (cert1), were significantly upregulated by Vh stimulation. Notably, Histone proteins (H1-like, H2A and H2B) (Supplementary Fig. S6D) and ubiquitin ligase were also upregulated, whereas the expressions of key molecule transporter proteins, solute carrier family (SLC2, SLC35, and SLC49), and sodium/potassium-transporting ATPase were downregulated by Vh stimulation (Supplementary Fig. S6D, Table S2).

### The inhibition of LD formation downregulated the expression of immune-related genes

Obscure puffer fish were treated with T863, a well-described DGAT1 inhibitor (Sui et al. 2023), and their ability to induce LDs was assessed. As shown in Fig. 5A, TAGs exhibited a sharp decrease from 11.11% of wet weight in the liver from control group, to 9.51% in the low-dose treatment group (0.125 mg) and 4.51% in the high-dose treatment group (0.25 mg, *P* < 0.05), indicating that the treatment of T863 significantly inhibits the TAG synthesis in liver. While no remarkable differences were detected between the control and T863 treatment group in head kidney (Fig. 5A). In addition, immunoblot analysis was performed using LD marker PLIN3. Compared with the control group, the indicative bands for PLIN3 significantly weakened under the T863 treatment. The high-dose treatment (0.25 mg) showed stronger suppression effect than the low-dose treatment (0.125 mg) (Fig. 5B). To assess whether LD induction following *V. harveyi* stimulation also follows a T863 inhibition mechanism, obscure puffer fish were treated with T863 after the *V. harveyi* stimulation. Compared with the control and *V. harveyi* treatment group, TAG contents in liver exhibited significant decreasement in the Vh + T863 co-treatment group (Fig. 5C). Similarly, indicative bands for PLIN3 significantly weakened in the Vh + T863 co-treatment group (Fig. 5D), indicating that T863 treatment decreased the induction of *V. harveyi* stimulation on the LDs formation. Next, the host immune responses against bacterial stimulation post-LDs reduced were examined. Compared with the control group, *V. harveyi* stimulation significantly enhanced the expressions of *NK-lysin*, *TNF14* and *ISG15*. While the expressions of all the examined genes (*NK-lysin*, *TNF14*, *ISG15*, IL-1, Mx1, and IFNγ) were significantly decreased in the Vh + T863 co-treatment group compared with that in the Vh treatment group (Fig. 5E). Similarly, the expressions of immune signaling pathway elements, such as *ATF6*, *cGAS*, *PKR1*, *PKR2*, *mTOR*, and *TNFRsf1a*, were significantly decreased in the Vh + T863 co-treatment group compared with that in the Vh treatment group (Fig. 5F), suggesting that inhibition of LDs formation by T863 treatment downregulated the expressions of innate immune-related genes, and thus, LDs should play critical roles in modulating immune response.

## Discussion

LDs are cellular organelles rich in neutral lipids, which is a huge attraction for many pathogens (Bosch et al. 2021). However, more recently, numerous studies have revealed that LDs accumulated rapidly upon pathogen infection, and it was proposed that host innate immunity rewires lipid metabolism and weaponizes LDs during bacterial infections (Bosch and Pol 2022). Here, we studied for the first time that the induction of LDs post-stimulation with marine pathogenic bacteria *V. harveyi* in obscure puffer *T. obscurus*, and performed a proteomic and functional study to investigate the dynamic changes and potential regulatory function of LDs during bacterial infection in fish.

Although there were reports that few bacteria were unable to trigger LDs formation (D'Avila et al. 2006), boosting evidences demonstrated that bacteria, virus, parasites, as well as components from bacterial cell wall (such as LPS, BCG or LAM) could induce LDs accumulation, and this phenomenon has been considered as a hallmark during inflammatory processes (Bosch et al. 2020; Castoldi et al. 2020; den Brok et al. 2018; Monson et al. 2021a,b; Wan et al. 2007). However, most studies are focused on the interaction between intracellular pathogens and host LDs (Roingeard and Melo 2017), and limited to mammalians and few model invertebrates (Anand et al. 2012; Barletta et al. 2016; Bosch et al. 2020). In this study, we analyzed for the first time that the infection with *V. harveyi*, which is a kind of facultative bacteria and threatens both for humans and marine animals (Xu et al. 2022), could induce LDs accumulation in the liver of *T. obscurus* in an in vivo setting. Consistent with previous studies in mammals, the number and size of LDs as well as the expression of LDs marker protein PLIN2, were significantly induced upon infection with live and killed *V. harveyi*, confirming the induction of *V. harveyi* on the LDs accumulation. Moreover, we observed that *V. harveyi* induced LDs formation via a dose- and time-dependent manner. This finding was also in accordance with prior studies in mammals which showed that the LPS treatment promoted LDs’ formation in a similar manner (Bosch et al. 2020; Pacheco et al. 2002). Notably, LDs number rapidly increased at 24 h, while the size of LDs apparently enlarged at 48 h post-stimulation, showing the dynamic change of LDs during the bacterial infection. This phenomenon of increased LDS size at 48 h may be more akin to fusion, which was similar with the previous studies on mosquitoes *Aedes aegypti*, where significant fusion of LDs occurred after pathogen invasion of the gut, with a significant increase in size (Barletta et al. 2016). In addition, it has been evidenced that LDs could be induced via multiple mechanisms (Olzmann and Carvalho 2019). Here, several genes involved in LD biogenesis, including neutral lipid synthesis and degradation, fatty acid synthase, and transport, were significantly regulated upon *V. harveyi* stimulation, demonstrating that *V. harveyi* infection induced LDs accumulation mainly via promoting neutral lipids synthesis. This in part corresponds to what Lei et al. (Lei et al. 2019) detected in grass carp head kidney cells, where LDs number and TAG contents were significantly upregulated stimulated by LPS or poly I: C, suggesting that the LDs formation might be an antibacterial defense strategy in the bony fish. Together, those findings collectively indicate that *V. harveyi* could induce LDs formation in a time- and dose-manner via regulating neutral lipid synthesis and degradation.

Proteomic analysis of LDs has produced a quantity of LDs proteins, which corroborating the function of LDs as well as evidencing that its function is conserved from bacteria to human (Bersuker et al. 2018; Krahmer et al. 2013; Sui et al. 2023; Yang et al. 2012). To investigate the dynamic changes and potential regulation mechanism of LDs during bacterial infection, LDs were isolated and performed proteomics studies. Apart from the LD proteomes in fish (Sun et al. 2023), most of the identified proteins in the present study have been reported in previous LD proteomes of yeast, Drosophila, and mammals (Bersuker et al. 2018; Bosch et al. 2020; Currie et al. 2014; Krahmer et al. 2013; Yang et al. 2012), supporting the conservative property of LDs from bacteria to humans. Briefly, similar with the previous reported LD proteomes, the LD proteome in the present study revealed that numerous LD regulatory scaffold proteins (PLIN2 and PLIN3) and TAG metabolism enzymes, which played critical roles during LD biogenesis. Other proteins common to LDs of other cells (Bersuker et al. 2018; Bosch et al. 2020) were also found in the LD proteome, indicating the confidence LD proteome of obscure puffer liver. Surprisingly, the proteome in the present study contained a large number of mitochondria-associated proteins, as well as ERs and peroxisome proteins, which was consistent with the findings in zebrafish and *Carassius auratus* (Sun et al. 2023), but was much higher than the ratio in mammalian LD proteomics (Bosch et al. 2020). On the one hand, this result might be because of the highly motility of LDs that LDs can closely associate with other cytoplasmic organelles/structures (Monson et al. 2021b; Pereira-Dutra and Bozza 2021; Wan et al. 2007). On the other hand, nascent LDs originate from the ER (Olzmann and Carvalho 2019). LD-associated proteome has been reported as dynamic and complex, the composition of LD housing proteins varied according to the metabolic state and activation of cells (Bosch et al. 2020; Olzmann and Carvalho 2019; Pereira-Dutra and Bozza 2021; Xu et al. 2018; Zhang and Liu 2019). In the present study, *V. harveyi* stimulation induced more than 200 LD-associated proteins significantly expressed. Most proteins participate in lipid storage as well as LDs biogenesis. Among of which myeloid derived growth factor (*mydgf*) exhibited significant upregulation upon bacterial infection, this finding was consistent with the previous reports that cytokines, chemokines, and growth factors were distributed on the newly formed LDs upon leukocytes’ activation (Wan et al. 2007). In addition, histones were found on LDs in a number of animal cells and tissues (Anand et al. 2012), and exhibited significant increase post *V. harveyi* stimulation. These results supported the hypothesis that LDs may have in host response to infection. Another prominent module, possessing functions related to the ubiquitin system, was also upregulated, which might be involved in the regulation of LDs’ turnover (Alberts and Rotin 2010). Of note, although *V. harveyi* stimulation induced the LDs’ accumulation in liver as well as a series of differentially expressed proteins, a few innate immune system-related components were enriched on the surface of LDs, which was significantly distinct from the results in mammals (Bosch et al. 2020). Ingenuity pathway analysis revealed an enhancement of oxidative phosphorylation, which have been demonstrated to selectively orchestrate tissue macrophage homeostasis (Wculek et al. 2023). In summary, these analyses suggested that numerous immunity-related proteins were found on the LDs, and *V. harveyi* stimulation modulated the component of LDs proteins.

Current studies revealed that LDs not only store lipids, but also participate in the host’s immune processes (Bosch and Pol 2022; Bosch et al. 2020; den Brok et al. 2018; Pereira-Dutra and Bozza 2021). The antibacterial activity of LDs could be induced upon pathogen infection which had been demonstrated in mammals (Bosch et al. 2020). Recently, studies in the zebrafish and Drosophila showed that embryos rich in LDs exhibited stronger resistance against bacterial infection (Anand et al. 2012). In this study, enhanced antibacterial activity of LDs post *V. harveyi* stimulation were first detected in fish, strengthening the critical role of LDs during the bacterial infection. Notably, Vh-LDs not only inhibited the grow of Gram-negative bacteria, but also inhibit the Gram-positive bacteria, showing a novel broad-spectrum immunity. In mammalians, LDs were proved to function during the host immune response by modulating the production of inflammation mediators. The inhibitor of DGAT1 T863 can effectively inhibit LDs formation both in mammals and fish (Knight et al. 2018; Monson et al. 2021a; Xiang et al. 2021). In the present study, we found that treatment with T863 had a strong effect on TAG levels and markedly abolished LDs formation in liver of obscure puffer, which was compliance with the previous studies (Sun et al. 2021; Xiang et al. 2021). Of note, LD downregulation was accompanied by significantly downregulated immune-related genes’ transcription, even under the stimulation of *V. harveyi*, which showing a strong connection between LD formation and the immune response.

In conclusion, these results in present study first showed that *V. harveyi* could induce LDs’ accumulation in the liver of obscure puffer mainly by promoting the synthesis of neutral lipids. Moreover, *V. harveyi* stimulation promoted the enriching of immune-related factors on the LDs surface, and the *V. harveyi* stimulated LDs display enhanced and broad-spectrum antibacterial activity. While the inhibition of LDs formation downregulated the expression of immune-related genes, highlighting the potential critical roles of LDs during the bacterial infection. The research results will deepen the understanding of LDs biology and host immune defense mechanism, shedding light on the new strategies for the development of anti-infective therapies, ultimately supporting the healthy and green development of the aquaculture industry.

## Supplementary Information

Below is the link to the electronic supplementary material.

Supplementary file1 (DOCX 2208 KB)

## Data Availability

Any information can be requested from the first author.
