# Supplementary material for: Emerging role of lipid droplets in obscure puffer immune response against Vibrio harveyi
Source: Mar Life Sci Technol. 2025 Mar 17;7(2):271–83. doi: 10.1007/s42995-025-00286-w (PMC12102014; doi:10.1007/s42995-025-00286-w)
Supplement: Supplementary file 1 — Supplementary file1 (DOCX 2208 KB) [file 42995_2025_286_MOESM1_ESM.docx]

**Supplemental information**

**Emerging role of lipid droplets in Obscure puffer immune response against *Vibrio harveyi***

Xiaorui Song ^1, 2^, Yaxing Yang ^2^, Nan Cui ^2^, Tianying Lei ^2^, Xingkun Jin ^1, 2^, Ying Huang ^1, 2^, Yan Shi ^1, 2^, Zhe Zhao ^1, 2,^ *

^1^ Jiangsu Province Engineering Research Center for Marine Bio-resources Sustainable Utilization, Hohai University, Nanjing 210024, China.

^2^ Department of Marine Biology, College of Oceanography, Hohai University, Nanjing 210024, China.

*Corresponding author. College of Oceanography, Hohai University, Nanjing 210098, China.

E-mail address: zhezhao@hhu.edu.cn (Z. Zhao).

ORCID: 0000-0002-1124-791X


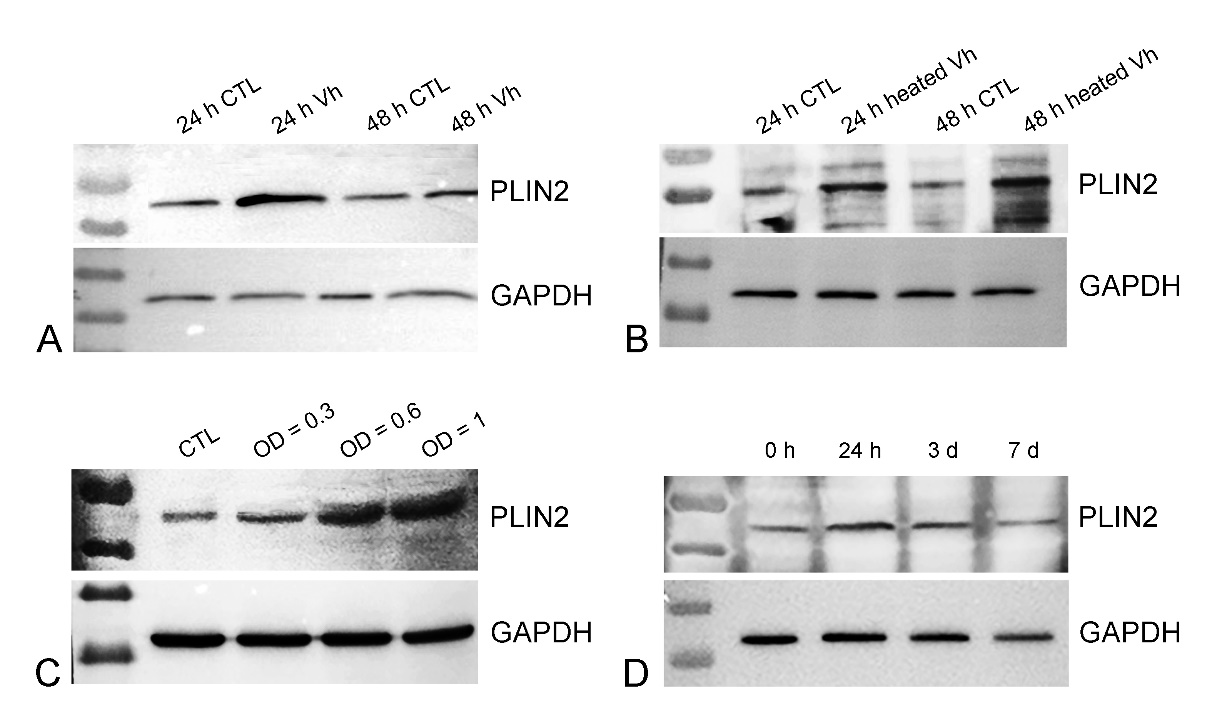


**Fig. S1**. *V. harveyi* stimulation induced Lipid Droplets accumulation in the liver tissue of obscure puffer.

(A and B) Western blot assay showed the expressions of the LD-resident protein PLIN2 in liver at 24 and 48 h post *V. harveyi* (OD=0.6) stimulation (A) and heat-killed *V. harveyi* (OD=0.6) stimulation (B), respectively.

(C) Western blot assay showed PLIN2 levels in response to different concentrations of *V. harveyi* stimulation (OD=0.3, 0.6, 1.0) in obscure puffer liver.

(D) Western blot assay showed PLIN2 levels at different time points (0 h, 24 h, 3 d, and 7 d) post *V. harveyi* stimulation (OD=0.6).


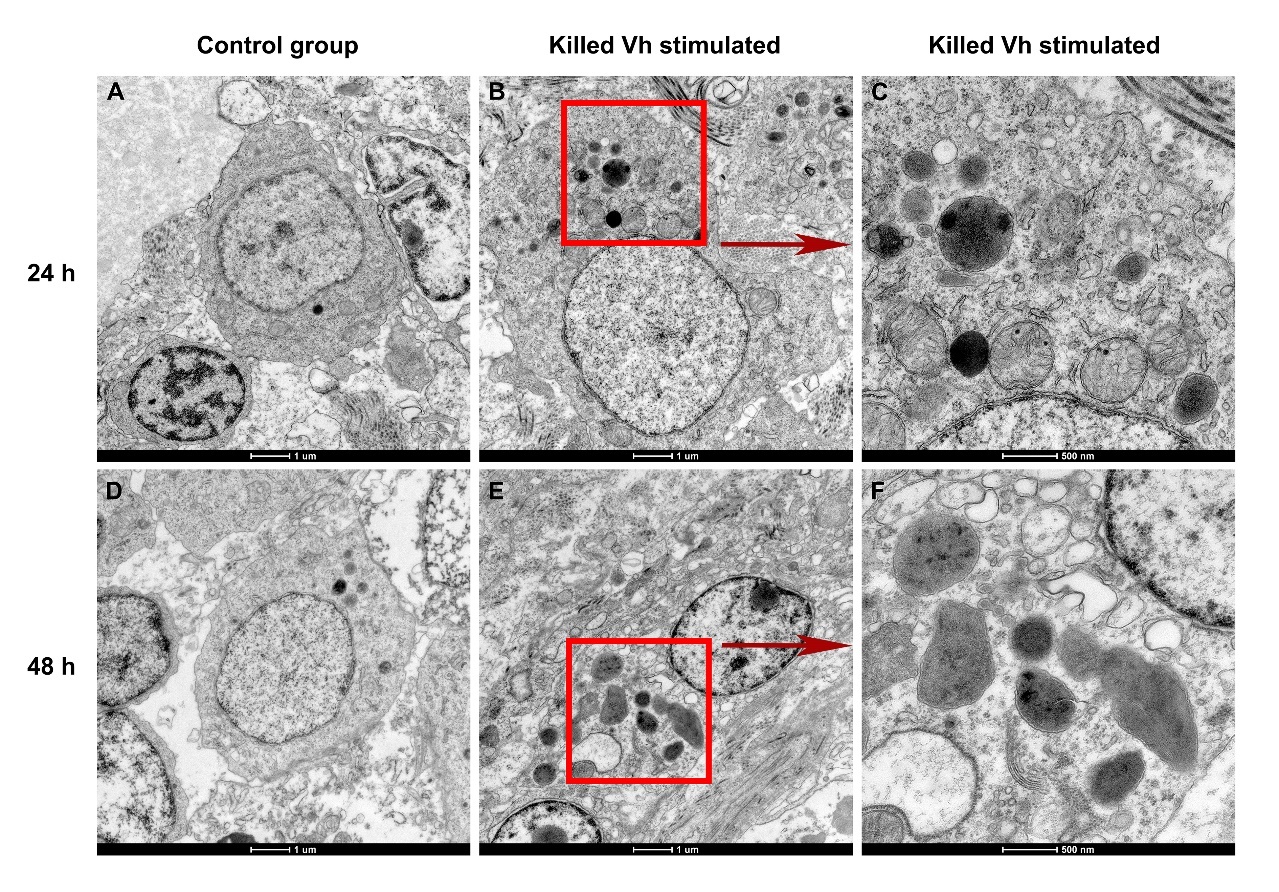


**Fig. S2**. Increasing cellular LD numbers in TEM images of heat killed *V. harveyi* stimulated liver.

(A and D) TEM images revealed fewer LDs in the liver from control group at 24 h (A) and 48 h (D), respectively;

(B and C) TEM images revealed increased cellular LD numbers in the liver at 24 h post *V. harveyi* stimulation (B), and the boxed area was enlarged in the (C).

(D and E) TEM images revealed increased cellular LD numbers and size in the liver at 48 h post *V. harveyi* stimulation (D), and the boxed area was enlarged in the (E).

Scale bar = 1 μm (A, B, D and E); Scale bar = 500 nm (C and F).


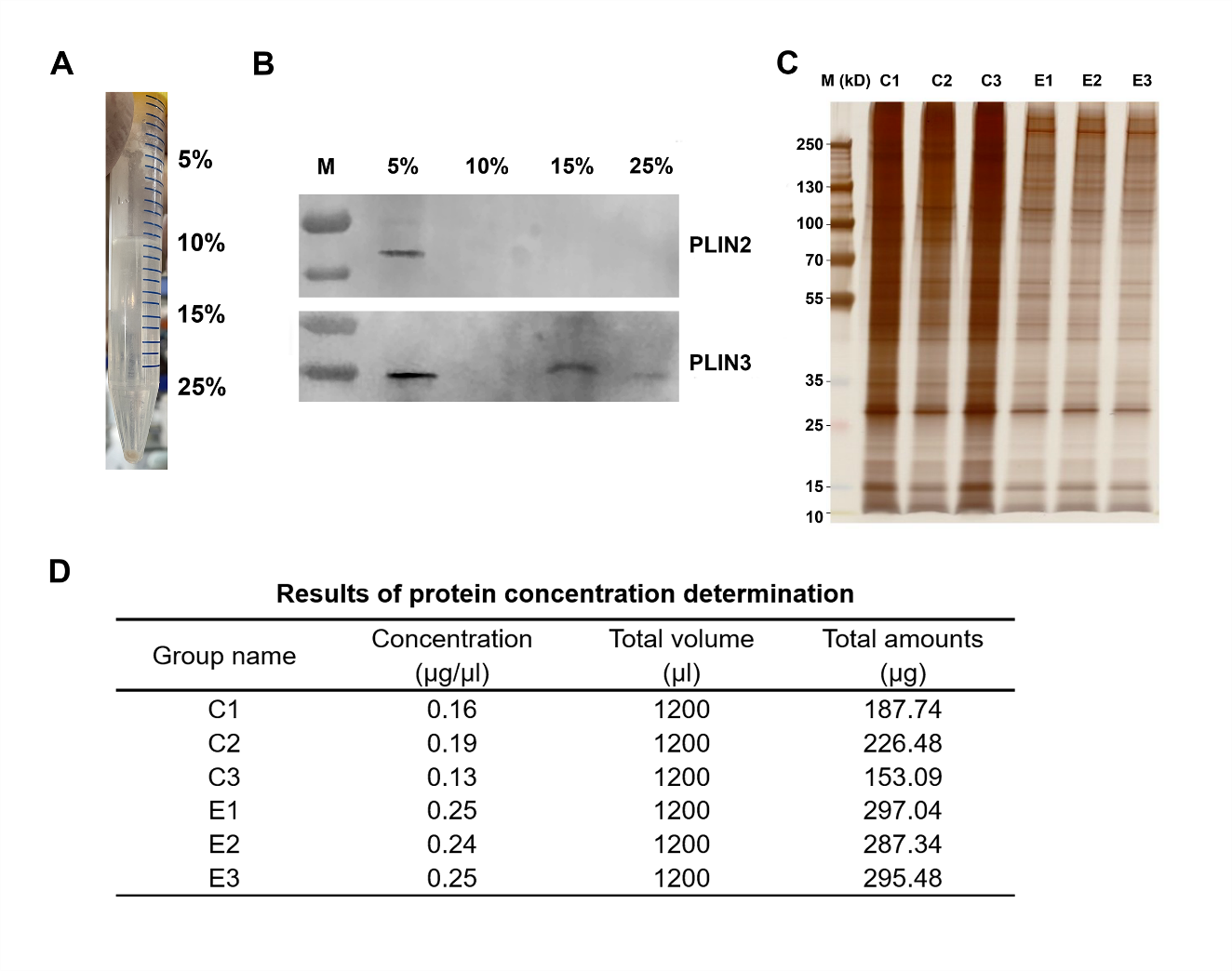


**Fig. S3**. Quality control of the isolated lipid droplets (LDs).

(A) The isolation of lipid droplets (LDs) via density gradient centrifugation;

(B) The validation of isolated LDs via Western blot;

(C) Isolated LDs proteins were separated by SDS-PAGE and then stained by silver staining;

(D) The concentration determination of isolated LDs proteins via BCA method.


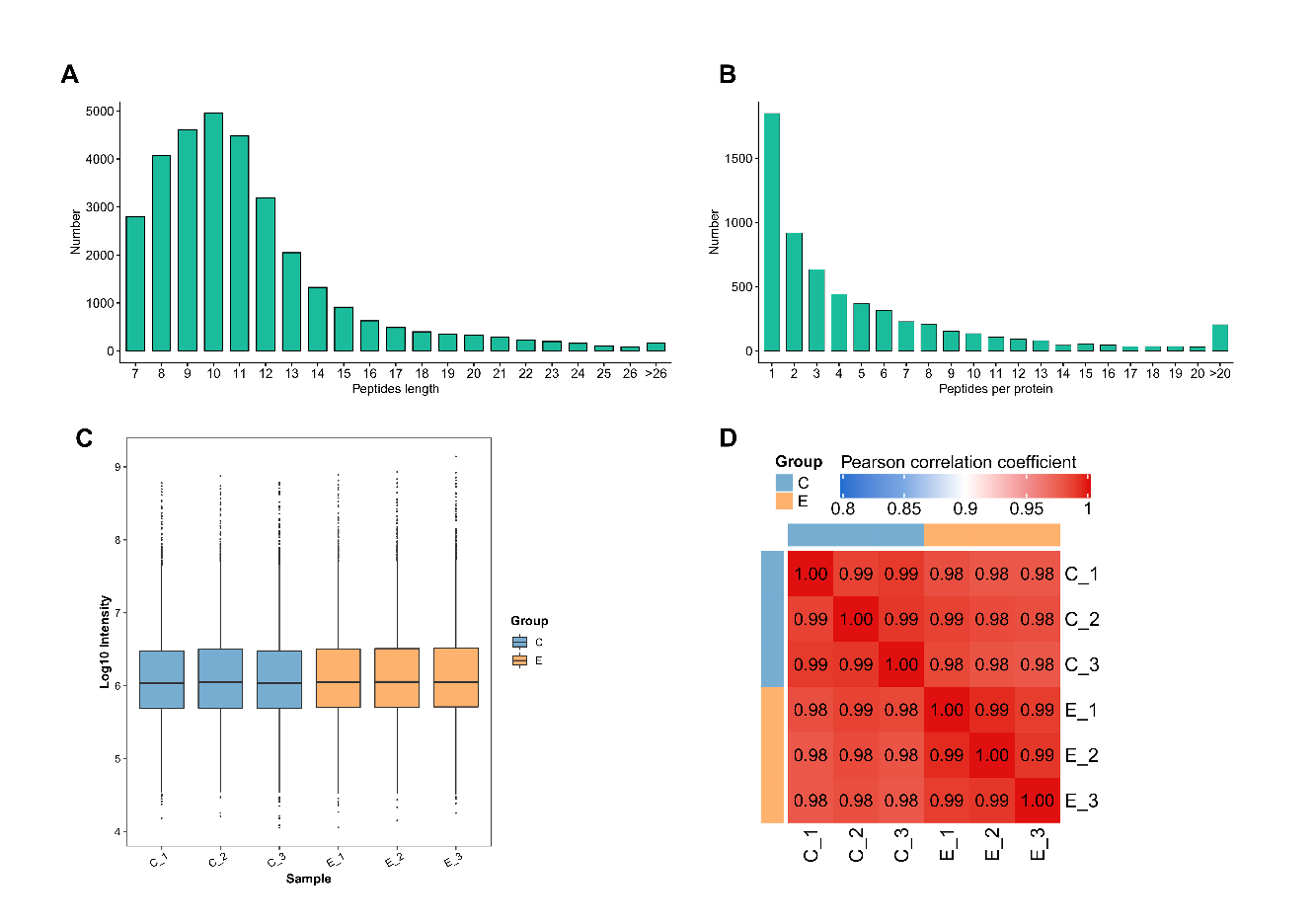


**Fig. S4**. Quality control of proteomic data results.

(A) The length distribution of identified peptides;

(B) The number distribution of identified peptides

(C) Box-plot of the Normalized intensity;

(D) Repeatability analysis *via* Pearson’s Correlation Coefficient;


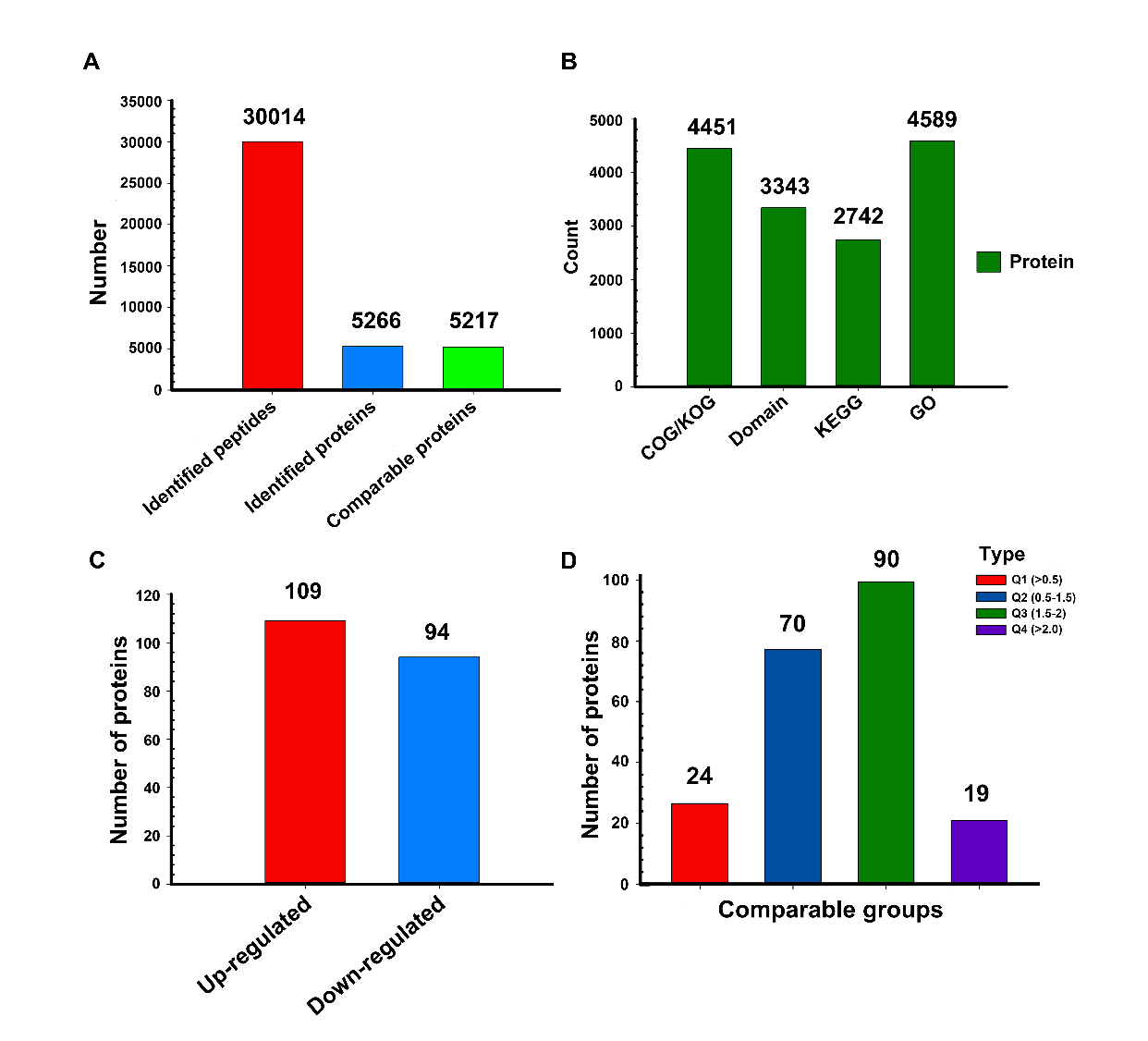


**Fig. S5**. Statistical information of proteomic data results.

(A) Identified peptides: The number of peptide sequences resolved by matching results; Identified proteins: The number of proteins resolved by specific peptide segments; Comparable proteins: The number of proteins quantified by specific peptide segments.

(B) The numbers of functional annotated proteins.

(C) Significantly changed proteins identified from comparison of control group and *V. harveyi* stimulated group.

(D) Differentially expressed proteins were divided into four clusters based on the changing tendencies and further analyzed by GO and KEGG enrichment.


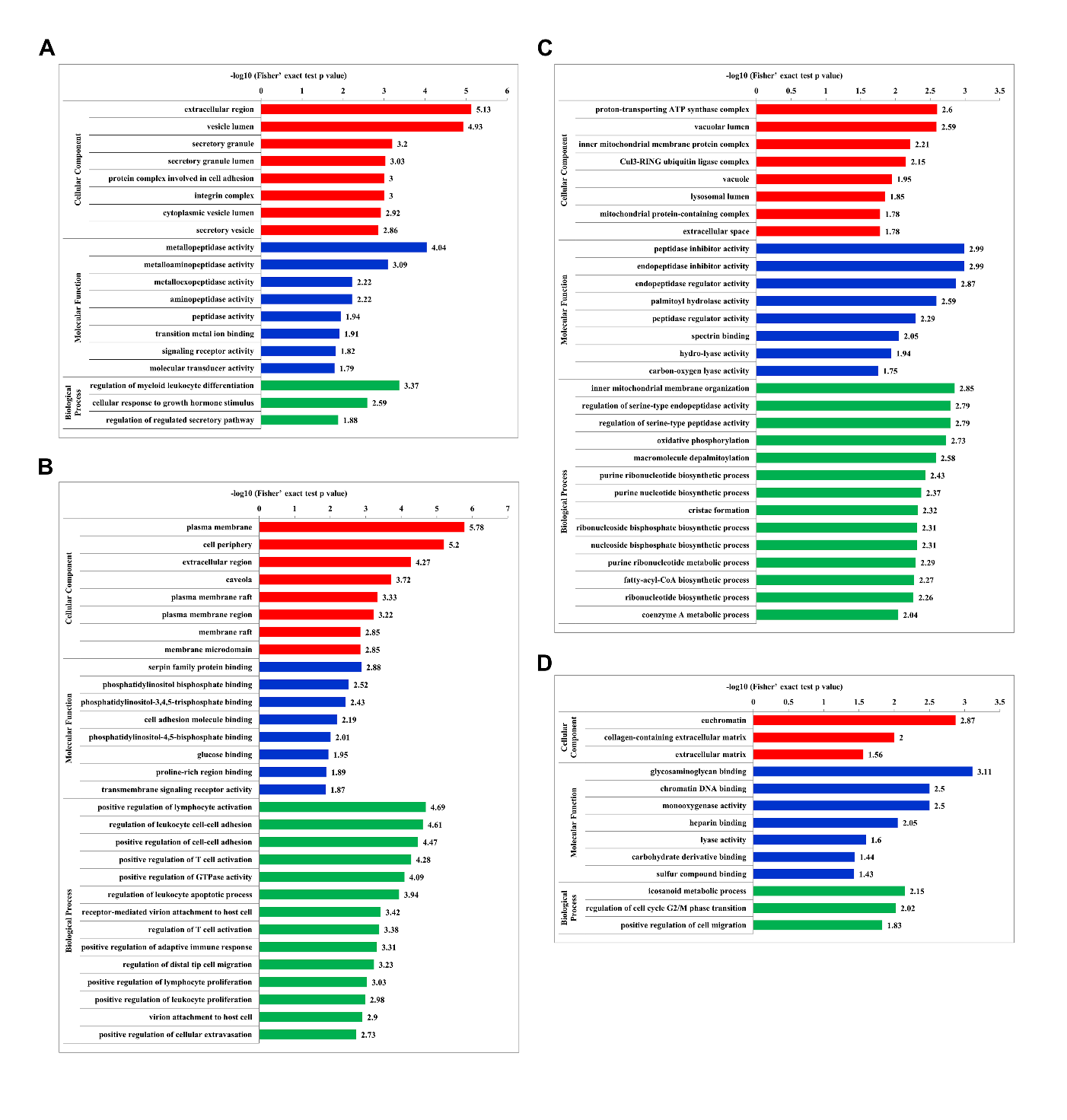


**Fig. S6**. Functional enrichment of four clusters based on the changing tendencies (A: Q1; B: Q2; C: Q3; D: Q4).

**Table S1. The primers used in the present study.**

| Gene name | Sequence（5’-3’） |
| --- | --- |
| *rt-FATP-*F | CGCCAGCGGCTACAGCAACTACA |
| *rt*-*FATP-*R | ATCCTGCCTCTTGGCTTTCGCTTGTCTGATT |
| *rt-CD36-*F | CCCGCTGGAGCCATCTTCATACCC |
| *rt*-*CD36-*R | TGTAACCCCACAGGAGTTCTTTGACCGT |
| *rt-ATP5D-*F | TCACGGGTACTTTCGGCATCCTTCC |
| *rt*-*ATP5D-*R | GCTGGTCCAAAGAAACCGCCTCCT |
| *rt-FASN-*F | CCCTCTTTCTTTGCCGCTGTCATTC |
| *rt-FASN-*R | TCTCCTTCAGTGTCTCAACCCATTTGTAGTC |
| *rt-ATGL-*F | ATGATGAAGAGGAGGCAGCGGTGGT |
| *rt-ATGL-*R | CAGAGCTTTGTGCAGGGTGGGTGAG |
| *rt-DGAT2-*F | ACTTCCGCCTGCCCGTCCTTC |
| *rt-DGAT2-*R | GCCGCTCCGCCGATGACAATG |
| *rt-DGAT1-*F | CGGTGCCCTCCGTGACCCCAGTT |
| *Rt-DGAT1-*R | GCCTCTTGGCTTTCGCTTGTCTGATT |
| *rt-ACSL-*F | TCCCTCATTGACAACATCTGTGCCTACG |
| *rt-ACSL-*R | GGTGCCCTTTACCTCCATTTGCTTCG |
| *rt-ACAT-*F | CTTAACACTCGCCACTCCAGCAACAACT |
| *rt*-*ACAT-*R | TGCCACCTGCCACCATCACATCC |
| *rt*-*NK-lysin-*F | CAACCTCCTCGATCCTGCTTCTGTG |
| *rt*-*NK-lysin-*R | TATGGCAAGTTCGTCCTGGTCTTCAT |
| *rt*-*TNF14-*F | AATCGCTATGCTGGGACTTGCTCTTC |
| *rt*-*TNF14-*R | CGACACGTTCTGGCACAAAGGATGAG |
| *rt*-*IL-1-*F | GCATCGTGACTCCTGACAGCATTCGT |
| *rt*-*IL-1-*R | TTCCACTTCATACTGGTGATTCCCTTC |
| *rt-ISG15*-F | TAATGTTAAAAATGGGGCGG |
| *rt-ISG15*-R | CAGGAACTGTTGAAGAAGAAGC |
| *rt-MX1*-F | TTGAAACCATCAAGCAACG |
| *rt-MX1-R* | TGTCCTGAGTGTAAACGAGCA |
| *rt*-*IFN-gR2*-F | GTCTTCTGTCACCATCCAGGG |
| *rt*- *IFN-gR2-*R | CCAGGATCCACGTCTGCTTG |
| *rt*-*ATF6*-F | CTTTCTACGTGGTGTCTTTTCGC |
| *rt*-*ATF6-*R | GTCTTTGTCCTGGATGATGCTGT |
| *rt*-*cGAS*-F | TGCAAAAGCGGCGGATGGA |
| *rt*-*cGAS-*R | CCAGACCAAACTAGTCCCCAATC |
| *rt*-*PKR2*-F | TCGCGCTCCAAATAACACG |
| *rt*-*PKR2-*R | CCAGACCAAAGTCCCCAATC |
| *rt*-*PKR1*-F | GGAAAGGAGATGGCTGTGAAG |
| *rt*-*PKR1-*R | AGTAGCGAACGATATTGGGGT |
| *rt*-*mTOR*-F | TGGAGTTTGAAGTGAAAAGGGC |
| *rt*-*mTOR-*R | TGCTGAAAGAAGAAAGTTGGGG |
| *rt*-*TNFRsf1a*-F | TCGGGGTGAGGGACACTGAGAT |
| *rt*-*TNFRsf1a-*R | TGCAAAAGCGGCGGATGGA |
| *rt*-*RS18*-F | CGCCCCGCCCAACTCGCCTGAATA |
| *rt*-*RS18-*R | CGAATGCCCCCGCCGTCCCTCTTA |

**Table S2. Proteins differentially expressed between control and *V. harveyi* stimulated LDs.**

| **Protein accession** | **Gene name** | **Regulated Type** | **E/C Ratio** | **E/C P value** |
| --- | --- | --- | --- | --- |
| A0A3B5JZH0 | ftsj3 | Up | 1.676223405 | 0.025064585 |
| A0A3B5KW15 | ANPEP | Down | 0.497017451 | 0.027725927 |
| A0A674NV52 | mtap | Up | 1.59444936 | 0.029126475 |
| H2S150 | lrp6 | Down | 0.584272525 | 0.002739337 |
| H2T087 | -- | Down | 0.485392418 | 0.003437597 |
| H2UUH3 | ACLY | Up | 1.522345574 | 0.004612821 |
| A0A3B5JUF9 | ATP1B1 | Down | 0.631324767 | 0.003623142 |
| A0A3B5JWY3 | tnfaip3 | Up | 1.634807986 | 0.007556312 |
| A0A3B5KHG6 | -- | Up | 1.606844117 | 0.001752313 |
| A0A3B5KHP7 | LOC101078132 | Down | 0.007236284 | 0.010936461 |
| A0A3B5KHZ8 | LOC101067429 | Down | 0.553080431 | 0.005339613 |
| A0A3B5KIF4 | LOC101076648 | Up | 1.826522064 | 0.017062452 |
| A0A3B5KJR5 | LOC101073513 | Down | 0.656790239 | 0.011973626 |
| A0A3B5KW58 | fxn | Up | 1.583780097 | 0.005376789 |
| A0A674MCM6 | LOC101074759 | Up | 1.615503474 | 0.000211922 |
| A0A674MFB8 | ndufb4 | Up | 1.641376179 | 0.002675854 |
| A0A674MFE8 | -- | Down | 0.660289384 | 0.030408069 |
| A0A674MJP8 | ank1 | Up | 1.87918561 | 0.030207526 |
| A0A674MKF4 | -- | Up | 1.641803863 | 0.029614284 |
| A0A674MT52 | -- | Up | 1.978463458 | 0.048348867 |
| A0A674MUV5 | LOC101072875 | Down | 0.474416339 | 0.006965071 |
| A0A674MYI6 | LOC101063861 | Down | 0.450186057 | 0.007668308 |
| A0A674MZR3 | cert1 | Up | 1.625616506 | 0.023321978 |
| A0A674N4G8 | plat | Down | 0.623742976 | 0.035873974 |
| A0A674N7X8 | atp5po | Up | 1.637502702 | 0.002487597 |
| A0A674N8U8 | -- | Down | 0.548469258 | 0.020457911 |
| A0A674N9G3 | FZD7 | Up | 1.738719648 | 0.026121542 |
| A0A674NDT2 | DOCK2 | Down | 0.641705694 | 0.004108812 |
| A0A674NE01 | -- | Down | 0.565203596 | 0.005662375 |
| A0A674NH30 | -- | Down | 0.522195218 | 2.77776E-05 |
| A0A674NQP9 | LOC101076989 | Down | 0.500418899 | 0.00052687 |
| A0A674NT03 | -- | Down | 0.593124734 | 0.000229141 |
| A0A674NUI9 | LOC101068611 | Up | 1.545466094 | 0.036029627 |
| A0A674NXQ6 | LOC101074990 | Down | 0.622890196 | 0.001929603 |
| A0A674NXT7 | LOC101069989 | Down | 0.500865676 | 0.000137092 |
| A0A674P2X6 | enpep | Down | 0.469936477 | 8.26252E-06 |
| A0A674P9H6 | -- | Up | 1.621649916 | 0.002304926 |
| A0A674PAP4 | LOC101075059 | Down | 0.661989609 | 0.009536241 |
| A0A674PAP8 | -- | Up | 2.295057181 | 0.00918735 |
| A0A674PAW9 | -- | Down | 0.606081427 | 0.027789984 |
| A0A674PB23 | LOC101079122 | Down | 0.483166118 | 0.000809132 |
| A0A674PN29 | -- | Down | 0.416410805 | 0.000101928 |
| H2RZL7 | -- | Down | 0.606238443 | 0.001493131 |
| H2S675 | prpf38a | Up | 2.601337376 | 0.015025206 |
| H2SJH5 | emc4 | Up | 1.526138493 | 0.019800719 |
| H2SJJ4 | timm9 | Up | 1.554676708 | 0.017732342 |
| H2SLN8 | LOC101069338 | Up | 1.55479075 | 0.031455316 |
| H2SPE6 | -- | Up | 1.755470131 | 0.037741686 |
| H2SY09 | marcks | Up | 1.570170973 | 0.035601787 |
| H2SZH3 | atp5pb | Up | 1.513474729 | 0.016649597 |
| H2T673 | LOC105416191 | Up | 2.471441309 | 0.000560872 |
| H2TXA1 | -- | Up | 1.636903777 | 0.014272499 |
| H2U054 | fkbp11 | Down | 0.633811706 | 0.001665217 |
| H2U6K2 | mpeg1 | Down | 0.531634508 | 0.00030203 |
| H2UDI5 | -- | Down | 0.45848764 | 0.01026689 |
| H2UHM1 | gosr2 | Up | 1.819922852 | 0.014564288 |
| H2UMH2 | AMDHD1 | Up | 1.662670789 | 0.006706158 |
| H2UZL7 | -- | Down | 0.542115899 | 0.032845003 |
| H2V3D0 | LOC445928 | Down | 0.419235208 | 8.08629E-05 |
| H2V5Y8 | LOC101070408 | Up | 1.879193125 | 0.004800742 |
| H2V8E4 | LOC101065526 | Down | 0.661583041 | 0.049869754 |
| H2VA54 | -- | Up | 2.733555197 | 0.005405074 |
| A0A3B5JYA0 | -- | Down | 0.188469741 | 9.327E-06 |
| A0A3B5JZ21 | -- | Up | 1.802818973 | 0.001139031 |
| A0A3B5K047 | -- | Up | 2.36854161 | 0.013809899 |
| A0A3B5K0Q6 | -- | Down | 0.578663745 | 0.038430745 |
| A0A3B5K0R0 | hpgd | Up | 1.59384829 | 0.023256067 |
| A0A3B5K126 | -- | Up | 2.047981371 | 0.019317239 |
| A0A3B5K242 | -- | Down | 0.439301129 | 0.007571714 |
| A0A3B5K2R6 | -- | Down | 0.489314189 | 0.003424676 |
| A0A3B5K390 | psme3 | Up | 1.75931727 | 0.005820185 |
| A0A3B5K3W6 | dpt | Up | 1.622934747 | 0.008194865 |
| A0A3B5K4R5 | tanc1 | Down | 0.621039602 | 0.003688435 |
| A0A3B5K7Y2 | -- | Up | 1.785105217 | 0.008462611 |
| A0A3B5KBM3 | -- | Up | 1.643309551 | 0.004317568 |
| A0A3B5KC29 | LOC101063900 | Down | 0.48246313 | 0.021187242 |
| A0A3B5KC33 | mblac1 | Down | 0.583374494 | 0.003521131 |
| A0A3B5KCB0 | retreg2 | Up | 1.585070316 | 0.033049167 |
| A0A3B5KG01 | -- | Down | 0.521433228 | 0.040232986 |
| A0A3B5KH04 | -- | Up | 1.851702312 | 0.007896687 |
| A0A3B5KH94 | mrpl12 | Up | 1.833281046 | 0.008609105 |
| A0A3B5KHE7 | LOC105416693 | Down | 0.640440284 | 0.003033776 |
| A0A3B5KIA3 | -- | Down | 0.651367902 | 0.047074589 |
| A0A3B5KIC3 | LOC105417307 | Up | 1.581989005 | 0.03916275 |
| A0A3B5KIH5 | LOC101068036 | Down | 0.564779198 | 6.09871E-05 |
| A0A3B5KK94 | LOC101063282 | Down | 0.588002361 | 0.040356835 |
| A0A3B5KKN5 | LOC101063230 | Up | 1.636656011 | 0.025065202 |
| A0A3B5KLB2 | -- | Up | 2.739861928 | 0.044589262 |
| A0A3B5KLP9 | pnisr | Up | 1.652206975 | 0.01258018 |
| A0A3B5KM71 | -- | Up | 1.50740418 | 0.040359268 |
| A0A3B5KMD4 | LOC101063034 | Up | 1.623284734 | 0.000724271 |
| A0A3B5KME6 | -- | Up | 2.234599628 | 0.031441453 |
| A0A3B5KND3 | -- | Down | 0.664066085 | 0.006321978 |
| A0A3B5KQK2 | -- | Up | 1.602431873 | 0.004357236 |
| A0A674M8T6 | -- | Up | 1.658718625 | 0.001065724 |
| A0A674M9E5 | -- | Up | 1.552997507 | 0.007587487 |
| A0A674M9M6 | -- | Up | 1.645282006 | 0.000657255 |
| A0A674M9Q4 | LOC101065028 | Up | 1.523902474 | 0.024095281 |
| A0A674M9R3 | RRM2 | Down | 0.51822796 | 0.007844983 |
| A0A674MD03 | dock8 | Down | 0.66455787 | 0.004834747 |
| A0A674MDB7 | pank1 | Up | 1.552805282 | 0.029089151 |
| A0A674MDF4 | -- | Up | 1.828266579 | 0.02228671 |
| A0A674MDI2 | -- | Up | 7.188094194 | 0.000316378 |
| A0A674MFI8 | -- | Down | 0.653978763 | 0.013949135 |
| A0A674MGY5 | -- | Up | 1.770708114 | 0.040031581 |
| A0A674MIY7 | -- | Down | 0.581828588 | 0.001537632 |
| A0A674MJU8 | -- | Down | 0.614799492 | 0.008503817 |
| A0A674MKJ5 | LOC115246418 | Up | 1.704347316 | 0.022231081 |
| A0A674MLA5 | txnrd2 | Up | 1.618542028 | 0.001091054 |
| A0A674MLI9 | UBQLN4 | Up | 1.569963039 | 0.013475577 |
| A0A674MPP4 | LOC101074478 | Up | 3.393872409 | 0.000504399 |
| A0A674MPT8 | -- | Down | 0.659844708 | 0.001128849 |
| A0A674MPX7 | ttc17 | Up | 1.970390472 | 0.012902574 |
| A0A674MQR0 | LOC115251740 | Down | 0.639332505 | 0.038524806 |
| A0A674MRH9 | -- | Down | 0.654791057 | 0.01259197 |
| A0A674MRM0 | LOC115252165 | Down | 0.529069427 | 6.31332E-05 |
| A0A674MSD2 | -- | Up | 1.941020086 | 0.009367992 |
| A0A674MSW3 | -- | Down | 0.573933351 | 0.000808264 |
| A0A674MSY7 | -- | Down | 0.648359252 | 0.010939963 |
| A0A674MU63 | -- | Down | 0.108452402 | 0.000850943 |
| A0A674MY11 | pdlim1 | Up | 1.739053445 | 0.003308975 |
| A0A674MYQ5 | lrmp | Down | 0.65804707 | 0.006606487 |
| A0A674MYX3 | -- | Down | 0.592218614 | 0.016745492 |
| A0A674MZY0 | LGALS4 | Down | 0.495531062 | 0.000351167 |
| A0A674N0P6 | klhl24 | Up | 1.558249084 | 0.006727079 |
| A0A674N130 | -- | Up | 1.784213706 | 0.000229186 |
| A0A674N3T3 | -- | Down | 0.56824484 | 3.64296E-05 |
| A0A674N526 | LOC101070585 | Down | 0.476557197 | 1.32602E-05 |
| A0A674N5U9 | -- | Up | 2.256905401 | 0.000367733 |
| A0A674N6F6 | -- | Down | 0.620388525 | 0.0024514 |
| A0A674N9A5 | -- | Up | 2.513786276 | 0.000166231 |
| A0A674N9S1 | atp5f1e | Up | 2.460335998 | 0.019199355 |
| A0A674NBL2 | -- | Down | 0.2477906 | 0.000538299 |
| A0A674NCV2 | ppt1 | Up | 1.50003671 | 0.018343757 |
| A0A674NCX0 | CPA1 | Up | 7.634996671 | 0.00578613 |
| A0A674ND02 | manf | Up | 1.659022693 | 0.000271534 |
| A0A674NIJ1 | gm2a | Up | 1.509610546 | 0.017321993 |
| A0A674NMA0 | -- | Up | 2.168787229 | 0.00274266 |
| A0A674NN41 | -- | Up | 1.562061291 | 0.031792663 |
| A0A674NNL6 | -- | Up | 3.197262266 | 0.000180856 |
| A0A674NQM7 | MGST3 | Up | 2.104820842 | 0.00997243 |
| A0A674NRM8 | arhgap45 | Down | 0.591327893 | 0.003298334 |
| A0A674NSZ7 | -- | Up | 1.621642247 | 0.043940764 |
| A0A674NV45 | -- | Down | 0.517243786 | 0.004289703 |
| A0A674NVK1 | LOC101072643 | Up | 1.674065947 | 0.037840325 |
| A0A674NW12 | -- | Up | 1.942640763 | 0.000570287 |
| A0A674P147 | -- | Down | 0.609928688 | 0.003969273 |
| A0A674P3R8 | LOC105418351 | Down | 0.587111928 | 0.00612705 |
| A0A674P7G4 | -- | Down | 0.478629801 | 0.004107486 |
| A0A674PBQ4 | -- | Down | 0.614029682 | 0.026882778 |
| A0A674PDN6 | -- | Up | 1.522042945 | 0.023281627 |
| A0A674PGY8 | LOC101065443 | Down | 0.552323576 | 0.037573276 |
| A0A674PHE9 | LOC101061415 | Up | 1.534510474 | 0.008162742 |
| A0A674PIM6 | LOC101062751 | Up | 1.622527587 | 0.008367424 |
| A0A674PJ07 | -- | Down | 0.597385192 | 0.000220285 |
| A0A674PR68 | -- | Down | 0.633739266 | 0.044656821 |
| F7VJJ3 | SSAT-1 | Up | 1.576132729 | 0.001049149 |
| H2RIQ0 | LOC105418898 | Up | 1.893434654 | 0.000596811 |
| H2RL96 | rbp1 | Down | 0.483353178 | 0.048300139 |
| H2RN33 | LOC105419432 | Down | 0.563771327 | 0.031285215 |
| H2RT92 | LOC101063769 | Down | 0.371471209 | 0.001474434 |
| H2RU31 | LOC101075287 | Up | 1.510287318 | 0.002053479 |
| H2RYC9 | -- | Up | 1.52471962 | 0.00231357 |
| H2RZR0 | ostm1 | Down | 0.571768409 | 0.031409674 |
| H2S040 | ITIH3 | Down | 0.639461631 | 0.024021878 |
| H2S518 | cavin2 | Up | 1.916117883 | 0.00864541 |
| H2S6Y3 | CDR2 | Down | 0.577713412 | 0.032112389 |
| H2S7R6 | LOC101065361 | Down | 0.642818703 | 0.00033365 |
| H2S812 | -- | Up | 1.537187516 | 0.016485944 |
| H2SEP7 | LOC101066271 | Down | 0.498359695 | 0.000327342 |
| H2SEZ4 | -- | Up | 1.704306444 | 0.000878359 |
| H2SH86 | -- | Down | 0.64337379 | 0.004056667 |
| H2SHM0 | SLC49A3 | Down | 0.487039652 | 0.011170935 |
| H2SK46 | -- | Up | 2.495579075 | 0.006018511 |
| H2SQH2 | rbmx | Up | 1.756309395 | 0.003322199 |
| H2SW72 | -- | Up | 1.502538428 | 0.040659645 |
| H2SXI6 | tspan8 | Down | 0.586325475 | 0.002308688 |
| H2T2P1 | LOC101064467 | Down | 0.598793724 | 0.004269144 |
| H2TF78 | LOC101075087 | Down | 0.562706099 | 0.025071353 |
| H2TG40 | -- | Down | 0.657379921 | 0.006037921 |
| H2TGR0 | sarnp | Up | 1.596801893 | 0.015858004 |
| H2TH19 | elmo3 | Down | 0.653567234 | 0.002290212 |
| H2TL02 | tmem201 | Up | 1.847705547 | 0.004006844 |
| H2TLW8 | LOC101062688 | Up | 1.86775606 | 0.023611671 |
| H2TQJ6 | LOC101076023 | Down | 0.561720381 | 0.004752837 |
| H2TRB6 | -- | Down | 0.558995471 | 0.000644068 |
| H2TXF7 | mydgf | Up | 1.613620424 | 0.000394787 |
| H2TYA7 | tmem59 | Up | 1.545955076 | 0.030124628 |
| H2U223 | LOC101065388 | Down | 0.536978687 | 0.001458619 |
| H2U3Y0 | snap29 | Down | 0.648209964 | 0.013613187 |
| H2U6Y5 | -- | Down | 0.646958787 | 1.82385E-05 |
| H2UCP5 | -- | Down | 0.296360379 | 0.000654304 |
| H2UEZ3 | -- | Up | 1.619134965 | 0.04132042 |
| H2UF39 | EPB41L2 | Up | 1.760478964 | 0.048416731 |
| H2UIR1 | -- | Up | 1.663401239 | 0.001797247 |
| H2UJG4 | eci1 | Up | 1.91716813 | 0.001991069 |
| H2UKI9 | LOC101069446 | Up | 1.730759998 | 0.023925061 |
| H2UNR5 | LOC101069841 | Up | 2.562794339 | 0.003250393 |
| H2V0V7 | pus7l | Up | 1.64577462 | 0.040205205 |
| H2V1G7 | spag7 | Up | 1.553582129 | 0.009651729 |
| H2V7L9 | -- | Down | 0.511975857 | 0.00075724 |
| H2VBT8 | LOC101061126 | Up | 1.563233309 | 0.026440838 |
| H2VCS7 | LOC101075700 | Up | 1.825855163 | 0.001162377 |
